# Tissue-resident immune cells: from defining characteristics to roles in diseases

**DOI:** 10.1038/s41392-024-02050-5

**Published:** 2025-01-17

**Authors:** Jia Li, Chu Xiao, Chunxiang Li, Jie He

**Affiliations:** Department of Thoracic Surgery, National Cancer Center/National Clinical Research Center for Cancer/Cancer Hospital, Chinese Academy of Medical Sciences and Peking Union Medical College, Beijing, China

**Keywords:** Immunological disorders, Tumour immunology, Innate immunity, Adaptive immunity

## Abstract

Tissue-resident immune cells (TRICs) are a highly heterogeneous and plastic subpopulation of immune cells that reside in lymphoid or peripheral tissues without recirculation. These cells are endowed with notably distinct capabilities, setting them apart from their circulating leukocyte counterparts. Many studies demonstrate their complex roles in both health and disease, involving the regulation of homeostasis, protection, and destruction. The advancement of tissue-resolution technologies, such as single-cell sequencing and spatiotemporal omics, provides deeper insights into the cell morphology, characteristic markers, and dynamic transcriptional profiles of TRICs. Currently, the reported TRIC population includes tissue-resident T cells, tissue-resident memory B (BRM) cells, tissue-resident innate lymphocytes, tissue-resident macrophages, tissue-resident neutrophils (TRNs), and tissue-resident mast cells, but unignorably the existence of TRNs is controversial. Previous studies focus on one of them in specific tissues or diseases, however, the origins, developmental trajectories, and intercellular cross-talks of every TRIC type are not fully summarized. In addition, a systemic overview of TRICs in disease progression and the development of parallel therapeutic strategies is lacking. Here, we describe the development and function characteristics of all TRIC types and their major roles in health and diseases. We shed light on how to harness TRICs to offer new therapeutic targets and present burning questions in this field.

## Introduction

**Fig. 1:**
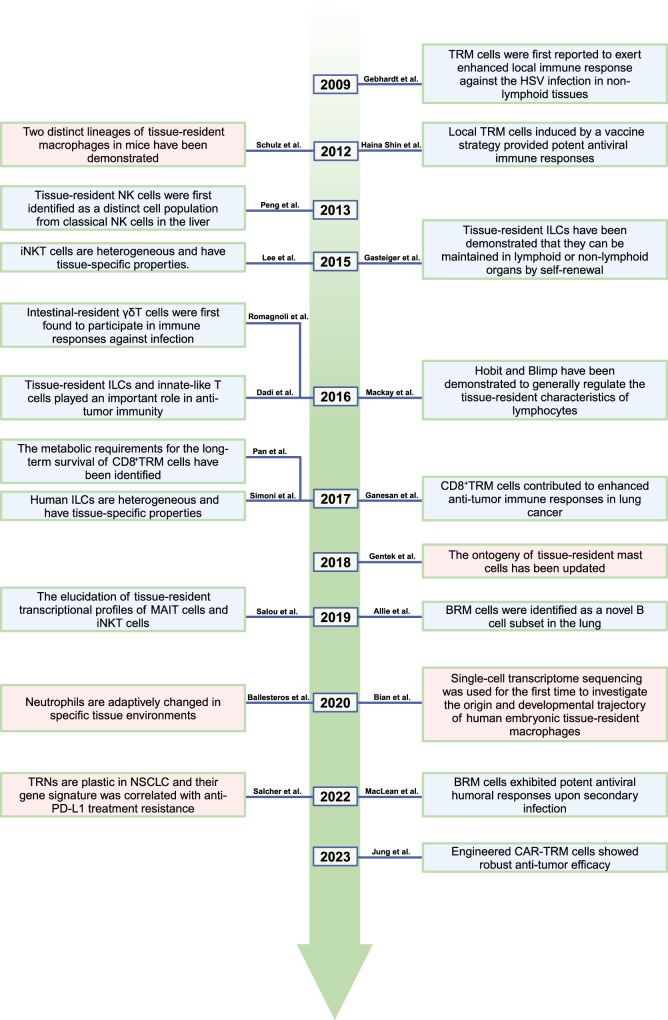
Milestone research of TRICs. Key events in the exploration of TRICs were retrospectively shown from 2009 to the present. The text box with a blue background depicts lymphoid-lineage TRICs, and the text box with a pink background describes myeloid-lineage TRICs. TRM tissue-resident memory T, HSV herpes simplex virus, ILC innate lymphoid cell, BRM resident memory B, MAIT mucosal-associated invariant T, iNKT invariant natural killer T, TRN tissue-resident neutrophil, NSCLC non-small cell lung cancer, PD-L1 programmed cell death-1-ligand-1, CAR chimeric antigen receptor, Blimp B lymphocyte-induced maturation protein, Hobit Homology of Blimp in T cells. This figure is created with BioRender.com

Many diseases are caused by immune dysfunction. The innate and adaptive immune systems coordinate to ensure immune surveillance, clearance, and homeostasis of organisms. Innate immune cells act as the first barrier line and contribute to motivating adaptive immune responses,^1^ while the adaptive immune cells usually circulate between different organs and perform specific immune responses.^2^ Interestingly, some immune cell populations from innate or adaptive immunity develop tissue-resident features and hardly perform circulation, and they often undergo specific phenotypic and functional reprogramming.^3–5^ Here, we used the term, tissue-resident immune cells (TRICs), to characterize these cell populations. Studies in the last 20 years have constantly explored each member of TRICs. Inspiringly, under the help of rapidly iterative technologies, their distinct origins, developmental trajectories, maintenance mechanisms, specific phenotypic and transcriptional characteristics, and functions are increasingly clear. Landmark studies on TRICs are shown in Fig. 1 (Fig. 1).

**Fig. 2:**
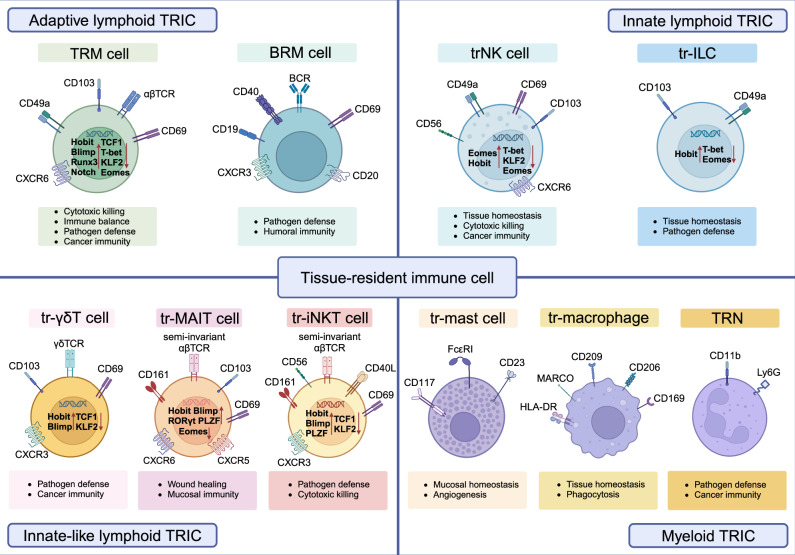
The types, representative markers, and fundamental functions of TRICs. The surface markers used for defining and identifying TRICs are shown. The markers include differentiation markers like CD4, CD8, and CD20, and tissue residency markers include CD103, CD49a, and CD69. Some critical chemokine receptors for tissue location and key TFs responsible for TRIC development are displayed. The transcriptional characteristics of BRM cells are undefined, and myeloid TRICs have distinct transcriptional regulation in different tissues that is difficult to summarize in one plot. The main functions of each TRIC in physiological and pathological conditions are listed. trNK tissue-resident natural killer, tr-ILC tissue-resident ILC, tr-mast cell tissue-resident mast cell, tr-macrophage tissue-resident macrophage, tr-γδT tissue-resident γδT, tr-MAIT tissue-resident MAIT, tr-iNKT tissue-resident iNKT, TCR T cell receptor, BCR B cell receptor, CXCR C-X-C chemokine receptor, CD the cluster of differentiation, HLA human leukocyte antigen, MARCO macrophage receptor with collagenous structure. This figure is created with BioRender.com

Lymphoid or myeloid-lineage immune cells can develop tissue-resident features and possess specific properties that circulating immune cells are not equipped with.^5,6^ We define these cells as lymphoid-lineage TRICs (hereafter lymphoid TRICs) and myeloid-lineage TRICs (hereafter myeloid TRICs) respectively. They extensively distribute in second lymphoid organs (SLOs) or peripheral non-lymphoid tissues, such as skin, brain, liver, lung, kidney, and gastrointestinal tract.^5^ Endogenous regulatory mechanisms- and environmental factors-inducible phenotypic reprogramming co-maintain the long-term persistence of TRICs in peripheral tissues and inhibit their detachment from tissues into circulation (Fig. 2).

TRICs are highly heterogeneous across different tissues and participate in many biological processes. They share some common functions with canonical circulative immune cells, including defense against pathogens and cell collaboration. Notably, long-term tissue residency endows some specific functions of TRICs, such as maintaining durable immune defense and local homeostasis.^7^ Nonetheless, the lasting tissue retention also has side effects, increasing the vulnerability to autoimmune disease, cancer, and graft rejection.^5,7,8^ Therefore, how to utilize TRICs to maintain conducive immune functions is an issue worth exploring.

With the growing applications of immunotherapies in many diseases, TRICs are thought to be a potential target for novel therapy development. People have begun to explore the feasibility of targeting disease-specific TRICs as therapeutic strategies.^9–14^ For example, some vaccines can induce the formation of CD8^+^ tissue-resident memory T (TRM) cells that defend against pathogen reinfection and amplify anti-tumor immunity.^15–17^ Furthermore, neutralizing antibodies blocking proinflammatory cytokines produced by TRICs can alleviate autoimmune disease progression.^18,19^ Therefore, a thorough understanding of TRICs is very essential for further therapy optimization and development.

In this review, we comprehensively describe the origins, biological characteristics, and functional evolutions of each TRIC type. We summarize their roles in physiological conditions and pathological contexts, covering the major diseases in which TRICs intervene. Moreover, we emphasize the potential of TRICs as promising therapeutic targets and predictive biomarkers for diseases. We hope the understanding of TRICs is helpful for future investigation of immunity in health and diseases.

## Definition of tissue-resident immune cells

### The origins of TRICs

**Fig. 3:**
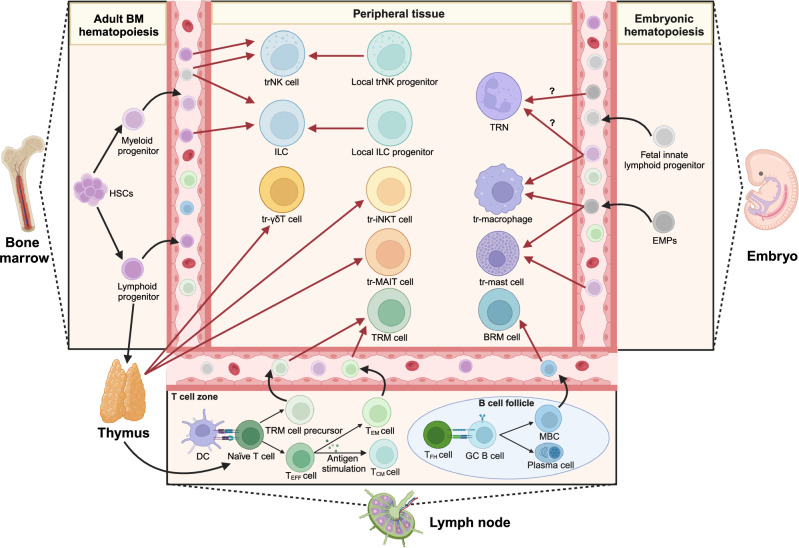
The developmental trajectories of TRICs. HSCs from bone marrow can differentiate into TRIC precursors. Embryonic hematopoiesis is another progenitor cell source for TRICs. Tissue-resident macrophages and mast cells originate from embryonic EMPs, and circulating monocytes or mast cell precursors can also acquire tissue-resident features after entering tissues. Most lymphoid TRICs develop postnatally and derive from lymphoid progenitors. Tissue-resident ILCs and trNK cells derive from fetal innate lymphoid progenitors during the embryonic period or postnatal lymphoid progenitors. Local progenitors can also differentiate into tissue-resident ILCs and trNK cells in some tissues. After leaving the thymus, tissue-resident MAIT cells, iNKT cells, and γδT cells localize in peripheral tissues, while naïve αβT cells migrate into SLOs. In SLOs, naïve T cells are activated by mature DCs, then T cells proliferate and differentiate into T_EFF_ and memory-like precursors. T_EFF_ will transform into memory T cells stimulated by cognate antigens, including T_CM_ and T_EM_ cells. TRM cells can derive from primary TRM precursors or T_EM_ cells. The interaction with T_FH_ cells promotes GC B cells to differentiate into memory B cells and plasma cells. During the effector stages, circulating memory B cells can become the precursor of BRM cells. BM bone marrow, EMP erythroid myeloid progenitor, HSC hematopoietic stem cell, DC dendritic cell, T_EFF_ effector T, T_EM_ effector memory T, T_CM_ central memory T, T_FH_ follicular helper T, GC germinal center, MBC memory B cell. This figure is created with BioRender.com

The precursors and original sites of TRICs are distinct. Some TRICs acquire tissue-resident properties during embryogenesis, such as tissue-resident macrophages and mast cells, tissue-resident natural killer (trNK) cells, tissue-resident innate lymphoid cells (ILCs), and γδT cells, while other TRIC types postnatally establish tissue residency during the effector stages (Fig. 3).^20,21^ TRICs have different lifetimes under different contexts. In most cases, TRICs achieve long-term tissue residence by self-renewal, but they also depend on the replenishment of circulating immune cells to varying degrees.^5,6,22^

Regarding myeloid cells, tissue-resident macrophages and mast cells can originate from both embryogenic precursors and adult hematopoietic stem cells (HSCs)-derived myeloid progenitors.^21,23–28^ They primarily rely on self-renewal for long-term persistence within tissues.^23,29–37^ Exactly, macrophages in different tissues have distinct requirements for the extra supplement of bone marrow-derived progenitors.^20,37–42^ Subsequently, regardless of their origin, they are plastic and develop tissue-specific phenotypes after entering tissues.^39,43–48^ Neutrophils are always short-lived in tissues. They can quickly develop tissue-specific behaviors under the steady state and rely on some factors to prolong their tissue-resident lifespan.^49–52^ These neutrophils with a relatively long-tissue livelihood are termed tissue-resident neutrophils (TRNs).

Among lymphoid cells, TRM cells are the most widely studied TRIC type. They derive from circulating memory precursor cells following T cell activation and develop tissue-specific phenotypes across different tissues.^53,54^ BRM cells may be derived from memory B cells (MBCs) triggered by pathogen infection during adulthood, and the inducible bronchus-associated lymphoid tissue (iBALT) may also specifically contribute to their formation in the lung.^55–58^ As for unconventional T cells, tissue-resident mucosal-associated invariant T (MAIT) cells and invariant natural killer T (iNKT) cells derive from postnatal precursors in the thymus, and they adopt tissue-resident characteristics after lineage commitment.^59–62^ In contrast, tissue-resident γδT cells derive from thymocytes during embryonic and adult periods, but they are endowed with tissue residency properties before exiting from the thymus and residing in peripheral tissues.^63–65^ Uniquely, the progenitors of resident ILCs distribute into peripheral tissues during the embryonic periods, such as the fetal intestine, liver, and tonsils. ILCs can also derive from progenitors in the bone marrow and local tissues during the adult period.^66–69^ Tissue-resident ILCs predominantly maintain themselves by self-renewal, with less role for circulating or locally derived hematopoietic progenitors in supporting ILC maintenance.^68–71^ Similarly, trNK cells differentiate from bone-marrow-derived progenitors, differentiated classical NK (cNK) cells in adulthood, or tissue-resident progenitors during embryogenesis.^66–68,72^

### Advanced technologies for TRIC finding

The identification of TRICs is subject to experimental technologies. Parabiotic surgery is the direct method to identify TRICs. People combine the circulation of two homogeneous mice, and the circulating cells will establish equilibrium in both mice, and tissue-resident cells do not enter circulation.^73,74^ TRM cells, ILCs, trNKs, BRM cells, tissue-resident macrophages, tissue-resident iNKT, MAIT cells, and TRNs have been identified via this approach.^30,38,49,56,62,68,75–79^ Intravascular labeling employs antibodies to distinguish blood-borne and tissue-resident lymphocytes that will not be labeled, like TRM cells and BRM cells.^56,80,81^ Moreover, flow cytometry and cytometry by time of flight are used to identify TRICs by detecting the specific marker profiles.^79,82–85^

High-throughput sequencing technologies help to construct the comprehensive fate and feature map of TRICs.^86^ Fate-mapping analysis and lineage tracing system contribute to clarifying their origins and developmental trajectories.^31,38,49,79,87^ T cell receptor (TCR) and B cell receptor sequencing analyses have been used to explore the immunological characteristics of TRM cells, γδT cells, and BRMs.^7,57,88^ Especially, single-cell RNA-sequencing (scRNA-seq) analysis is effective for exploring the phenotypic or transcriptional profiles of TRM cells, BRM cells, trNK cells, tissue-resident macrophages, TRNs, and ILC2s in physiological states and even diseases.^49,55,57,69,83,89–99^ In this way, trNK cells are found to express distinct cytokine receptors from cNK cells with heterogeneous phenotypes across the liver, lung, and salivary glands.^83,100–104^ In addition, three distinct tissue-resident macrophage subsets with different transcription profiles in tumor tissues were also identified by scRNA-seq.^40^ In disease studies, scRNA-seq analysis of patients’ samples confirmed the existence of TRICs in multiple sclerosis (MS),^105^ autoimmune nephritis,^106^ influenza infections,^107^ and cancers.^84^

### The phenotypic markers and transcriptional profiles of TRICs

The phenotypes of TRICs are deeply shaped by molecules and cellular interaction within the local tissue environment, so the same kinds of TRIC render to acquire high heterogeneity in different organs.^22,82^ Characteristic phenotype markers and regulatory transcription factors (TFs) are useful in distinguishing them (Fig. 2).

CD69, CD103, and CD49a (very late antigen-1, VLA-1) are canonical tissue-resident markers for lymphoid TRICs, but their expression varies across different tissues. Most TRICs, including TRM cells, tissue-resident ILCs, and γδT cells, predominantly express CD103 in mucosal tissues. However, in non-mucosal tissues, CD103 expression is not common or even absent, while CD69 and/or CD49a are more expressed in these cell populations.^108,109^ A combined expression of CD40L, CD56, CD161, and CD69 characterizes iNKT cells,^110,111^ MAIT cells are defined by CD161, C-C motif chemokine receptor (CCR) 5, and CCR6.^60^ BRM cells generally express CD69 and CD45RB,^89^ and trNK cells express CD69, CD49a, and C-X-C chemokine receptor (CXCR) 6.^112^ In addition to those markers, most lymphoid TRICs upregulate genes encoding tissue adhesion- or tissue recruitment-related molecules accompanied by downregulating genes that induce them to detach tissues, such as sphingosine 1 phosphate receptor-1 (S1PR1) and CCR7.

Lymphoid TRICs share similar transcription profiles. Several TFs are essential for the formation and development of lymphoid TRICs. First, Krüppel-like factor 2 (KLF2) regulates the tissue egress of lymphocytes and T cell homeostasis by increasing S1PR1 and CD62L expression.^101,113^ Low KLF2 is a unique feature in lymphoid TRICs, such as CD8^+^TRM cells, CD4^+^TRM cells, trNK cells, and iNKT cells.^101,114–117^

B lymphocyte-induced maturation protein (Blimp) and Homology of Blimp in T cells (Hobit) are indispensable for the maintenance of TRM cells, iNKT cells, and liver-resident NK cells.^101,118^ They repress the expression of circulation-promoting factors KLF2, S1PR1, CCR7, and TCF1 in lymphoid TRICs.^74^ Notably, they exhibit functional complementarity.^101,119^ Lacking either Hobit or Blimp partially inhibits the persistence of CD8^+^TRM cells and iNKT cells in tissues, but the absence of both greatly suppresses the formation of these TRICs.^101,118,120–122^ In contrast to Hobit and Blimp, T-bet and Eomesodermin (Eomes) are negative regulators for TRM cells. The forced expression of either T-bet or Eomes impairs CD8^+^TRM cell formation.^123,124^ Other TFs also contribute to the TRIC development, including aryl hydrocarbon receptors for skin CD8^+^TRM cells and liver-trNK cells,^125,126^ and Notch for lung CD8^+^TRM cells.^127^

However, unlike lymphoid TRICs, tissue-resident macrophages, TRNs, and mast cells do not have identical molecular features, which may be due to myeloid TRICs developing natural tissue-resident properties during embryogenesis.^22,82^

### Cellular interactions involving TRICs

The interactions between TRICs and other cell components in tissues are important for the tissue residency of TRICs and their functions.^128^ The interaction mechanisms include the direct binding of membrane surface receptors and ligands such as CD103 expressed on TRICs binding to E-cadherin on epithelial cells to ensure the tissue maintenance of TRICs,^129^ and cell communication mediated by secreted cytokines. For example, CD4^+^TRM cells can regulate the production of tumor necrosis factor-α (TNF-α) in CD8^+^TRM cells and promote the maturation of dendritic cells (DCs).^70,130^ IL-2 from CD8^+^TRM cells enhances the cytotoxic abilities of NK cells.^130^ In addition, the multilateral cellular interactions can lead to more complicated immune effects. Alveolar macrophages (AMs) guide CXCR3^+^lung-BRM cells to secondary infected sites by inducing the expression of CXCL9 and CXCL10 by other immune cells.^131^ CD69^+^CD4^+^lung-resident helper T cells promote the local retention of BRM cells and CD8^+^TRM cells after primary viral clearance, thus developing protective immune responses against secondary infection.^132^ Tissue-resident mast cells can induce fibroblast infiltration and interact with neutrophils to mediate wound repair and inflammatory processes.^133–137^ Furthermore, tissue-resident mast cells even participate in adaptive immune responses by serving as antigen-presenting cells (APCs) and recruiting and activating B cells and T cells.^137–139^

### Overview of TRIC functions

TRICs perform essential regulatory effects on local tissues. Under physiological conditions, the fundamental functions of TRICs include maintaining tissue repair and homeostasis, defending against pathogens, and regulating local immune responses.

The canonical tissue homeostasis-related TRICs are cutaneous MAIT cells, tissue-resident macrophages, and mast cells. MAIT cells promote wound healing.^140^ Microglia, the brain-resident macrophages located in the central nervous system (CNS), contribute to brain homeostasis.^141^ Tissue-resident mast cells also mediate intestinal mucosal homeostasis and tissue remodeling.^142–145^ Liver-resident NK cells suppress liver fibrosis by removing the pathological hepatic stellate cell-derived myofibroblasts.^146^ Tissue-resident γδT cells secret the growth factor amphiregulin to mediate tissue repair.^147,148^ Even in the heart, CCR2^−^heart-resident macrophages express high levels of growth factors and extracellular matrix (ECM) to mediate tissue remodeling.^149^

Given their long-term tissue residency, TRICs are frequently exposed to pathogen stimulation. So it is rationally thought that TRICs have enduring immune defense capability.^7^ Studies have proven this issue in many cases, including CD8^+^TRM cells against herpesvirus (HSV), respiratory syncytial virus, and influenza infection,^150–152^ CD4^+^TRM cells against HSV-2,^116^ and trNK cells against mouse cytomegalovirus and influenza infection.^153,154^ BRM cells also provide durable protective responses against influenza virus infection by producing neutralizing antibodies,^55^ and tissue-resident mast cells eliminate parasites and bacteria to achieve tissue defense.^139,155–159^ TRICs mainly secret proinflammatory cytokines, including TNF-α, interferon (IFN)-γ, and interleukin (IL)-2 to kill pathogens and expand tissue inflammation.^160–162^

TRICs keep local immune balance and prevent excessive inflammation mainly through expressing inhibitory factors or immune checkpoint molecules. For example, TRM cells, γδT cells, and trNK cells have been found to express the inhibitory immune checkpoints in many tissues, such as programmed death receptor-1 (PD-1), cytotoxic T lymphocyte-associated antigen-4 (CTLA-4), T cell immunoglobulin and ITIM domain (TIGIT), lymphocyte-activation gene-3 (LAG-3), and T cell immunoglobulin domain and mucin domain-3 (TIM-3).^112,163–165^

In contrast to the tissue-adaptive functions of lymphoid TRICs, myeloid TRICs with compartmentalized distribution always have tissue-specific functions, especially macrophages. For example, human fetal microglia contribute to regulating neuron differentiation.^99,166,167^ Intestine-resident macrophages and mast cells can positively regulate blood vessel integrity, intestine motility, and the enteric nervous system.^168–172^ Bone-resident macrophages (Osteoclasts) participate in remodeling bone tissues and the progression of genetic skeletal disease,^173,174^ and bone-resident mast cells have been found to regulate bone metabolism.^175,176^ Moreover, Kupffer cells localized in the liver secret hepatocyte growth factors to promote liver regeneration.^177^ Fat-associated macrophages facilitate fat storage and control energy loss.^73,178–180^

In conclusion, each type of TRIC has both genetic and adaptive tissue-specific functions. Their dysregulation could be the pathogenesis of certain diseases.

## Characteristics of lymphoid-lineage tissue-resident immune cells

Lymphoid-lineage TRICs include TRM cells, tissue-resident unconventional T cells, tissue-resident ILCs, trNK cells, and BRM cells. These lymphoid TRICs have effector functions and regulatory mechanisms similar to their circulative counterparts, whereas it is more interesting and significant to investigate the specific characteristics of tissue-resident lymphocytes.

### Tissue-resident memory T cells

Based on the expression profiles comprising lymphocytes homing receptor CD62L, chemokine receptor CCR7, memory T cell marker CD45RA and CD45RO,^181^ and co-stimulatory receptor CD28, memory T cells are divided into CD45RA^−^CD45RO^+^CD62L^−^CCR7^−^CD28^+^effector memory T cells (T_EM_) and CD45RA^−^CD45RO^+^CD28^+^CCR7^+^CD62L^+^central memory T cells (T_CM_).^182–186^ TRM cells are thought to be differentiated from primary memory precursors or circulating precursor T_EM_ with low killer cell lectin-like receptor subfamily G member 1 (KLRG1) expression, which undergoes activation and localization in peripheral tissues during the effector stage.^187–194^

TRM cells display distinct phenotypes from T_EM_ cells and T_CM_ cells. They highly express CD103, CD69, and CD49a, the representative markers of tissue residency, instead of expressing CD62L and CCR7.^110,195–197^ In brief, the classic TRM cell marker combination is CD45RA^−^CD45RO^+^CD103^+/^^−^CD69^+/^^−^CD49a^+/^^−^CCR7^−^CD62L^−^KLRG1^−^S1PR1^−^CD4^+^/CD8^+^. CD103 is the α subunit of αEβ7-integrin, binding to E-cadherin and helping T cells home to the epithelium and keep residence.^195,198^ Many studies have found CD103^+^CD8^+^TRM cells in epithelial tissues including skin,^199,200^ lung,^201^, and intestine.^202^ They are also in the SLOs, such as the spleen and lymphoid nodes.^3–5^ However, not all TRM cells express CD103, such as some CD4^+^TRM cells,^200,203^ so CD103 cannot be used alone to identify TRM cells. C-type lectin CD69 is an agonist of S1PR1 that is important for lymphocyte egress,^70^ thus CD69 negatively regulates the recirculation of T lymphocytes.^204^ Most TRM cells have a high level of CD69 and a low level of S1PR1.^197,205^ CD49a also plays an important role in the retention and survival of memory T cells in peripheral tissues.^206,207^

Parabiotic surgery shows circulating T cells of two congenic mice that are conjoined by the skin and exchanged blood will reach equilibrium within one week, whereas the resident T cells remain in the original position.^73,208^ But this approach cannot exclude the influence of local inflammation on circulating memory T cell recruiting.^209,210^ In vivo labeling by fluorochrome-conjugated antibodies combined with confocal imaging can also be used to distinguish the TRM cells and circulating T cells.^211^ However, this method is not fully suitable for identifying TRM cells in the red pulp of the spleen and liver sinusoids.^80^ Tissue transplantation can also be used to identify TRM cells, despite being restricted to surgical technologies.^22^ Parabiosis or in vivo labeling is not suitable for human TRM cell identification, but high-throughput sequencing technologies are available.^91–94^

With the regulatory support of intracellular mechanisms and local tissue environments, TRM cells undergo adaptive differentiation and keep tissue residency.^212,213^ First, epigenetic mechanisms regulate the expression of resident-related molecules, as the assay for transposase-accessible chromatin using sequencing (ATAC-seq) showed that the transcription start site region of *Itage* (encoding CD103) was accessible in TRM cells from the small intestine and salivary gland, as did the transcription start site of *Ccr9* (encoding CCR9) in intraepithelial lymphocytes TRM cells.^214^

In addition, many animal studies showed that TF Hobit, Blimp-1, Notch, and Runx3 were essential for the differentiation and maintenance of TRM cells in the lung, small intestine, and salivary gland.^101,127,214–216^ Runx2 and Runx3 also induce the cytotoxic functions of CD8^+^TRM cells.^217^ TF Bhlhe40 preferentially regulates the fitness and function of CD8^+^TRM cells by programming mitochondrial metabolism.^218^ In contrast, Eomes and T-bet suppress TRM cell differentiation.^124,219^ Interestingly, TFs involved in regulating TRM cells perform tissue-specific modulation. For example, Blimp deficiency greatly blocks the formation of lung-CD8^+^TRM cells but hardly affects skin-CD8^+^TRM cells.^49,101^ However, most results and conclusions are based on animal models. The transcriptional regulations of human TRM cells require further explicit investigation.^101^

Transforming growth factor (TGF)-β, IL-33, and TNF are potent external cytokines that enhance tissue residency of TRM cells. These TFs inhibit TRM cell migration by downregulating KLF2 and S1PR1,^114,220,221^ and TGF-β also induces CD103 expression on TRM cells.^179,193,222–225^ IL-15 and IL-7 are also crucial for maintaining skin-TRM cells. Deficiency in either breaks the local immune homeostasis, inhibiting TRM cells and impairing their responses in contact hypersensitivity.^226^ In addition, TRM cells also secrete cytokines to modulate biological processes which will be described later.^130,227^

Chemokines/chemokine receptors are also involved in regulating the location and functions of TRM cells, including CCR6, CXCR3, CCR5, CCR8, CCR9, and their ligands.^228^ As the tissue-homing molecule, CXCR6 is widely expressed in TRM cells. CXCR6 recognizes CXCL16 produced by endothelial cells in the lung and skin mucosa to promote TRM cells to keep retention in these tissue sites.^229,230^ In addition, the CXCL10/CXCR3 axis is pivotal for HSV-specific CD8^+^TRM cell differentiation and increment in trigeminal ganglia.^150^ High CXCR3 is also upregulated on CD8^+^TRM cells in the liver.^162^ CCR9 expression promotes CD8^+^TRM cells to remain in the small intestine epithelium where the ligand chemokine ligand (CCL) 25 is abundant.^231^ The CCR9/CCL25 axis also contributes to CD103^+^CD8^+^TRM cell differentiation.^232^ CCR8 maintains TRM cells in human skin.^233^ These findings suggest the importance of chemokines in maintaining the tissue residency of TRM cells, thereby influencing their functional adaptation.

Antigen stimulation is another essential motivator for TRM cell priming,^124^ and the sites of stimulation greatly determine the location of TRM cells.^211^ For example, influenza virus vaccines effectively induce the aggregation of CD4^+^TRM cells and influenza-specific CD8^+^TRM cells within lung tissues, thereby conferring durable immune protection.^234^ Other localized viral infections and even house dust mite infections also induced CD103^+^CD8^+^TRM cells to differentiate and reside in the brain and skin in mice models.^235–237^ However, some studies indicated that antigen stimulation was not always required for CD8^+^TRM cell establishment, and even the persistent antigen stimulation inhibited the expression of CD103 in local T cells.^70,224^ Therefore, the roles of antigen stimulation in regulating TRM cell formation need to be further confirmed.

TRM cells express inhibitory exhaustion molecules to maintain immune homeostasis, including PD-1, CTLA-4, TIM-3, and LAG-3.^238^ These negative regulators can attenuate the immune responses induced by TRM cells to avoid overreactions. These checkpoint molecules are targets of immune checkpoint blockades (ICBs), suggesting that TRM cells can respond to ICB therapies.^239–241^

### Tissue-resident unconventional T cells

Unconventional T cells (also termed innate-like T cells, ILTCs) are developed in the thymus.^59^ They are mainly involved in immune responses in non-lymphoid tissues, including iNKT cells, γδT cells, and MAIT cells.^59,242^ These ILTCs have TCRs with less diversity and recognize antigens without limitation of major histocompatibility complex (MHC)-related molecules,^242^ so they can make quick responses early when encountering antigen stimulation.^243^

Most γδT cells exist in the epithelium and mucosal tissues, and only a small number of γδT cells are present in circulation.^244^ Unlike TRM cells, tissue-resident γδT cells derive from double negative thymocytes during the embryonic period or postnatal thymus and directly seed into barrier tissues after maturing and exporting from the thymus,^63,245^ including skin,^246^ liver,^247^ and intestine epithelium.^248^ The known functions of tissue-resident γδT cells include defending against infection, maintaining homeostasis, and repairing tissues.^248^

iNKT cells develop in the thymus after birth. TCR coordinating with CD1d molecules expressed by CD4^+^CD8^+^double-positive thymocytes promotes the development of iNKT cells.^249–251^ iNKT cells express lymphocyte function-associated antigen-1, intercellular adhesion molecules-1, and tissue-resident molecule CD69.^74,252^ Given the different TF profiles and cytokine secretion, iNKT cells can be classified into three subtypes, iNKT1, iNKT2, and iNKT17 cells.^111,253^ T-bet regulates the development of IFN-γ-producing iNKT1 cells, while iNKT2 cells are regulated by GATA3 and mainly produce IL-4 or IL-13, independently of RORγt regulation. iNKT17 cells that produce IL-17 are regulated by RORγt.^254,255^ Additionally, BTB-zine finger TF, PLZF is also important for iNKT cell differentiation, and PLZF-deficient iNKT cells downregulate the secretion of granzyme B (GZMB), IL-4, IFN-γ, and TNF-α upon stimulation.^256–258^

Following the lineage commitment of iNKT cells, Hobit and Blimp-1 drive their tissue-resident transcriptional programs.^122^ iNKT cells show highly heterogeneous in peripheral tissues such as the skin, liver, lung, and adipose tissues without recirculating in the blood or lymphoid tissues.^62,259–266^ They can initiate a rapid immune response at the early stage of pathogenic lipid stimulation presented on CD1d and produce cytotoxic molecules or cytokines like IFN-γ, IL-17, and TNF-α. They also activate other immune cells, such as DCs and CD8^+^T cells, and further amplify the immune response.^110,267^ Therefore, they are one of the potent contributors to support barrier immunity.^243,259,260^

MAIT cells develop in the thymus after birth.^60,61,268^ Notably, iNKT cells and MAIT cells acquire tissue-resident transcriptional profiles before leaving the thymus.^62^ MAIT cells express CD161 and tissue-homing receptors, including CCR5 and CCR6, and they always aggregate in mucosal tissues to exert innate-like immune functions via non-TCR signals.^269^ However, the tissue-specific regulation of MAIT cells and γδT cells needs to be further explored, and it is still unknown how tissue-resident unconventional T cells renew in tissues.

### Tissue-resident innate lymphoid cells

The development of ILCs and NK cells shares some conserved processes between mice and humans. However, human ILCs exhibit greater heterogeneity and NK cells are highly specialized with distinct transcriptional regulation and functions.^67,270^ In both species, innate lymphoid progenitors generally differentiate into NK cell precursors and common helper innate lymphoid progenitors. Then, NK cell precursors give rise to cNK cells that usually exist in circulation.^270,271^ However, human NK cells display more functional and tissue-specific diversity, including the CD56^bright^ and CD56^dim^ NK cell subsets with distinct cytotoxic functions, as well as trNK cells like decidual and liver NK cells, which are less prominent in mice.^270^ Common helper innate lymphoid progenitors further differentiate into innate lymphoid cell precursors that generate helper-like ILCs, including ILC1s, ILC2s, and ILC3s, and another progenitor that generates lymphoid tissue inducer.^70,71,271–273^ In humans, ILC subsets are more phenotypically and functionally diverse than in mice. For instance, ILC3 subsets show great heterogeneity, including NKp44^+^ and NKp44^−^populations, which are less defined in mice.^270,274^ ILCs do not express antigen receptors but regulate innate immunity and tissue remodeling.^243,275,276^

Helper-like ILCs have specific cytokine-sensitive pathways and intrinsic regulatory TF profiles, which serve to modulate their differentiation processes.^275^ ILC1s are induced by IL-12 and IL-18, regulated by the T-bet, and mainly produce IFN-γ and TNF-α to mediate immune defense against intracellular pathogens.^277^ ILC2s develop in response to IL-25 and IL-33, and their dominant TF is GATA3. ILC2s secrete cytokines associated with the Th2 population, including IL-4, IL-5, IL-9, and IL-13, to promote tissue repair and maintain tissue homeostasis.^278^ RORγt regulates ILC3s development and ILC3s produce IL-17, IL-22, TNF, and granulocyte-macrophage-colony-stimulating factor.^70,279–282^ ILC3s are important for defending extracellular bacteria and are associated with autoimmune diseases.^67,283–285^

According to a parabiosis investigation, the majority of helper-like ILCs are found in host SLOs and peripheral tissues, indicating their tissue-resident characteristics.^68^ Most helper-like ILCs can maintain themselves by self-renewal in the physiological states but depend on the supplement of adult bone marrow-derived or local precursors in a few cases.^68–71^ For example, upon acute infection, tissue-resident ILC2s proliferate locally and still keep self-maintenance. The hematopoietic progenitors partially supplement the tissue-resident ILC2s pool under inflammation resolution and tissue repair.^68^ Therefore, the constitution of the tissue-resident ILCs pool is not identical under different conditions.

### Tissue-resident NK cells

NK cells are the core effector of innate immunity.^286^ The typical phenotypic markers of human NK cells are CD56 and CD16, and murine NK cells express NK1.1, NKp46, and CD49b.^287^ There are two NK cell subtypes, cNK cells, and trNK cells, entirely different in developmental trajectories, phenotypes, locations, and functions.^83^

Splenic cNK cells originate from bone marrow-derived progenitors in a T-bet and Eomes-dependent manner.^75,76^ trNK cells derive from various progenitors. They can originate from tissue-resident progenitors during the embryonic and adult stages, bone marrow-derived progenitors during adulthood, or even differentiated cNK cells.^66–68,72^ For example, liver-trNK cells originate from hepatic hematopoietic progenitor cells or hepatic stem cells.^70,75^ Lin^−^Sca-1^+^Mac-1^+^fetal-liver-derived hematopoietic precursor cells tend to develop into liver-trNK cells in the adult liver.^72,288^ Moreover, a study found that CD62L^+^Eomes^+^cNK cells could enter infectious sites and differentiate into Tcf1^+^CD69^+^trNK cells upon acute infection in the skin. Therefore, liver-trNK cells can maintain themselves in a bone-marrow-independent manner during adulthood. The mechanisms of renewal and maintenance of trNK cells in other organs are poorly understood.

trNK cells are widely distributed across many non-lymphoid tissues, such as the uterus, lung, intestine, skin, liver, adipose tissue, and salivary gland.^76,153,273,289–292^ Most of them express canonical markers of tissue residency, including CD69, CXCR6, and CD49a, whereas CD103 expression is not constitutive.^76,293,294^ The phenotypic and transcriptional profiles of trNK cells are highly heterogeneous within different tissues.^85,96^ Human liver-trNK cells can express CD56, CD69, CD49a, and CXCR6. Hobit and Blimp are the main TFs that regulate liver-trNK cell development.^100–102^ Lung-trNK cells express CD69 but selectively express CD49a or CD103.^177,295^ trNK cells located in the salivary gland upregulate both CD49a and CD49b, and T-bet and TGF-β signaling are indispensable for their differentiation.^103,104^ trNK cells in the uterine express CD94, NKG2A, CD49a, and CD103, and Eomes regulates their activation.^296,297^ Moreover, trNK cells downregulate the expression of lymphocyte egression markers, such as KLF2, S1PR1, and SELL. Some trNK cells also express immunosuppressive molecules PD-1, CTLA-4, and TIGIT.^83^

Therefore, the categories, localized development, and functions of trNK cells provide us with a better understanding of NK cell-involved immunity. However, their immune responses to stimulation especially those different from cNK cells need to be further studied.

### Tissue-resident memory B cells

Naïve B cells migrate to SLOs from bone marrow and mature after interacting with CD4^+^follicular helper T cells and DCs, and then mature B cells differentiate into plasma and MBCs upon antigen stimulation. MBCs will differentiate into effective cells and offer humoral immune responses to antigen stimulation.^298^ The long-tissue residency characteristics of MBCs are reported in many studies. Influenza-specific B cells that highly express CD69 and CXCR3 in the lung can persist in the same sites for a long time after the primary infection and immediately differentiate and produce antibodies against reinfection.^55,299,300^ Researchers also found a novel subset of MBCs that express CD69 and CD45RB were predominant in the gut and spleen but did not exist in the blood.^89^ CD45RB is associated with MBC differentiation, and CD45RB^+^B cells can localize outside germinal centers.^301^ Therefore, CD69^+^CD45RB^+^MBCs are recognized as a unique subset of MBC cells, defined as BRM cells.^89,302,303^ BRM cells have distinct features from non-resident MBCs. Similar to TRM cells, BRM cells downregulate the expression of CD62L and S1PR1, whereas they upregulate CD69 and CXCR3.^55,57,89,304^

The origins of BRM cells in tissues are still unclear. A study revealed that GC in lymphoid organs, instead of iBALT, is the source of most lung-BRM cells,^55,57^ and the small percentage of MBCs in the lung originating from iBALT had a distinct phenotype from other BRM cells.^55,305^ Studies demonstrated that BRM cells form where B cells mediate immune responses to infection.^56^ Intranasal administration of the influenza virus can induce the recruitment of CXCR3^+^circulating MBCs by CXCL9 and CXCL10 to infected lung tissues, and these MBCs will develop into immunoglobulin A-producing BRM cells in the respiratory mucosa.^90^ Pneumococcus infection also induces CD69^+^BRM cells in the lung.^58^ Therefore, BRM cells may born in B cell-induced immune responses.

According to a recent study, BRM cells can be classified into two categories based on their antigen specificity and B cell receptor repertoire, termed “bona fide” BRMs and “bystanders” BRMs. The former cells have a special affinity for viruses that activates them to differentiate and function. In contrast, bystander cells cannot recognize viruses directly.^304^ Intriguingly, a study found that BRM cells can transiently migrate after the secondary infection in the lung. The chemokines secreted by other infiltrating immune cells recruit BRM cells to infected regions and amplify localized immune responses.^131^ However, the roles of BRM cells in other tissues or pathological status are unclear.

## Characteristics of myeloid tissue-resident immune cells

Myeloid-lineage leukocytes perform innate immunity and regulate adaptive immune activity. Myeloid TRICs, including tissue-resident macrophages, TRNs, and tissue-resident mast cells, have been found in many tissues and organs.^20,49,79^ Here, we systematically summarize their development, maintenance, phenotypes, and functions.

### Tissue-resident macrophages

Tissue-resident macrophages are a highly heterogeneous subgroup. Macroglia in the brain, Kupffer cells in the liver, AMs in the lung, nerve- and airway-associated macrophages in the lung mesenchyme, Langerhans cells in the skin, fat-associated macrophages in the adipose tissues, red pulp macrophages in the spleen, large peritoneal macrophage in the peritoneum, and osteoclasts in the bone are widely studied.^169,306^ The canonical markers of human classical macrophages are CD11b and HLA-DR.^307^ Different tissue-resident macrophages have specific phenotypic profiles, such as AMs express CD11c, CD14, CD206, and CD169,^308^ Kupffer cells express resident markers CD163, CD32, and macrophage receptors with collagenous structure but do not express CD169,^309,310^ as well as interstitial macrophages express HLA-DR and CD14.^311,312^ The functions of tissue-specific resident macrophages are also highly tissue-adaptive. In brief, they are responsible for tissue homeostasis, tissue repair, tissue remodeling, and regeneration.^313^

Some macrophages originate from yolk-sac or fetal-liver embryonic progenitors before HSC formally appears, and others originate from circulating monocytes derived from HSC in adults.^21,23–25,314^ Embryonic progenitors-derived macrophages are a distinct cell population from canonical macrophages in the mononuclear phagocyte system with specific developmental trajectories. There are three main hematopoiesis waves during the embryonic period of mice.^25,315^ The primary hematopoiesis was around the embryonic day (E) 7.5, which produces early erythroid myeloid precursors (EMPs). Early EMPs can further differentiate into C-Myb^−^CD45^+^F4/80^bright^CD11^blow^ macrophages, which can migrate to the brain to form microglia.^316–318^ Brain microglia have been thought to originate exclusively from early yolk-sac-derived progenitors.^31,98^ Around E8.5, the second wave produces late EMPs. Late EMPs will colonize the fetal liver. Fetal-liver progenitors give rise to many kinds of tissue-resident macrophages, including Kupffer cells, Langerhans cells, AMs, etc.^319,320^ The third wave originated from HSCs produced in the aorta-gonad-mesonephros region at about E10.5, and these HSCs were transplanted into the fetal liver and differentiated into a variety of lineage blood cells, including CD45^+^F4/80^low^CD11b^high^macrophages/monocytes.^321,322^ Furthermore, cardiac-resident macrophages can derive from progenitors in both primitive and definitive hematopoiesis during embryogenesis.^323^

Many studies indicated that the postnatal tissue maintenance of yolk-sac or fetal-liver-derived macrophages in the brain, liver, spleen, lung, peritoneal, pancreas, and BM are not or minimal dependent on the replenishment of bone marrow-derived HSC and circulating monocytes.^23,29–32,78,79,324^ However, sometimes HSC-derived monocytes do constitute significant parts in resident macrophage pools.^38,39^ Circulating monocyte-derived macrophages will acquire identical tissue-specific characteristics, functions, and the ability of long-term self-renew as embryonic progenitors-derived macrophages, such as in the lung and liver,^31,39,43–46^ which fully manifest the plasticity of monocytes. For example, a study found that the Kupffer cell niches would be replaced by monocyte-derived macrophages that were phenotypically and transcriptionally similar to embryonic progenitors-derived Kupffer cells after depleting embryonic progenitors-derived Kupffer cells by diphtheria toxin (DT) in the mice model.^39^ In the lung and bone, circulating monocyte-derived macrophages are also found.^169,313^ Furthermore, a study using scRNA-seq analysis, parabiotic experiment, and fate-mapping analysis defined three tissue-resident macrophage subpopulations in the liver, kidney, heart, brain, and lung, based on their representative gene markers and the way they maintain themselves in tissues.^40^ One macrophage subtype TLF^+^TIMD4^+/^^−^LYVE1^+/^^−^FOLR2^+/^^−^ macrophages hardly depend on monocytes for maintenance, whereas another two macrophage types MHCII^hi^TIMD4^−^LYVE1^−^FOLR2^−^CCR2^−^macrophages and TIMD4^−^LYVE1^−^FOLR2^−^ CCR2^+^macrophages depend on monocyte-derived macrophage for renewing to varying degrees.^40,41^ In cancer, infiltrating monocytes and tumor-associated macrophages (TAMs) also express tissue-resident markers.^311,325,326^ Initially, the yolk-sac-derived macrophages were thought to be replaced by Ly6C^hi^ monocyte-derived macrophages and were not present in the adult intestine.^41^ However, a study found the existence of self-maintaining gut-resident macrophages during the embryonic period, and they were not completely replaced by monocyte-derived macrophages after birth. This sample further confirms the complementarity between two macrophages with different origins.^327^ Hence, at least a partial population of monocyte-derived macrophages can be categorized as tissue-resident macrophages.

We think tissue-resident macrophages are composed of different ratios of embryonic and monocyte-derived macrophages, which is associated with the accessibility and availability of corresponding tissues. Take microglia as an example, which solely originates from yolk-sac-derived progenitors due to macrophages derived from the fetal liver and monocytes being impeded by the blood-brain barrier.^31,328^

The transcriptional profiles of tissue-resident macrophages in different tissues are determined by their lineage-determining factors and environmental signals.^313^ SALL1, SALL3, and MEIS3 mainly regulate microglia, and in Langerhans cells, it is RUNX3 and ID2 that do. Both of the two tissue-resident macrophages are regulated by TGF-β produced by epithelial cells and neurons.^329–332^ In AMs, colony-stimulating factor (CSF) 2 and TGF-β control PPARγ expression. In bone, the expression of NFATC1 in osteoclasts is regulated by RANKL, OPG, and TGF-β.^329–334^

The study of human macrophages poses significant challenges, primarily due to the lack of macrophages in early embryos and the exceeding difficulty in isolating specific macrophages. The study findings based on animal models cannot be confirmed in humans. Encouragingly, transcriptomics is utilized as a research method in this field. Many studies using scRNA-seq have disclosed the origin and lineage of macrophages, including the detailed single-cell profile of human embryonic macrophages, which clarified the origin of resident macrophages in many tissues.^98^ scRNA-seq also assists people in exploring novel macrophage populations.^98,99,335^ In addition, spatial transcriptomics, proteomics, and organoid cultures are useful in deciphering the functions and crosstalk of resident macrophages in tissues.^336–339^

### Tissue-resident neutrophils

Embryonic neutrophils derive from definitive hematopoiesis,^49,79^, and adult neutrophils originate from HSCs in the bone marrow and migrate into tissues to provide immediate immune responses.^340^ The existence time of neutrophils in tissues is relatively short, which may explain why people pay less attention to how neutrophils acquire tissue-specific properties and their efforts for tissue residency.^341,342^

However, short-lived neutrophils depend on different mechanisms to reside in tissues and show distinct functional phenotypes.^343^ The analyses using mass cytometry and high-throughput sequencing have identified CD11b^+^Ly6G^+^neutrophil clusters that acquired tissue-specific phenotypes in different organs, including bone marrow, spleen, blood, skin, intestine, and lung. Regardless of the short time (about one day) that these neutrophils exist in tissues,^49^ neutrophils entering tissues also show plasticity in adapting to local environments. The results of parabiosis and transfer experiments indicated that neutrophils would quickly adopt similar characteristics as the cells that were initially present in this tissue after migrating into tissues.^49^ Therefore, we named those neutrophils with similar tissue-resident phenotypes to other TRICs as TRNs.

Neutrophils in the lung are the representative example of TRNs. Lung-resident neutrophils possess distinct transcription profiles and surface markers that help distinguish them from bone marrow and blood-circulating neutrophils.^51^ For example, neutrophils isolated from the lung express higher CD11b and CXCR4 but lower CD62L than neutrophils derived from the blood and bone marrow. They also upregulate the mRNA expression levels of Cd14, Plet1, and Sca1/Ly6a, which demonstrate how they differ from other origins-derived neutrophils.^51^ In addition, many factors within lung tissues promote neutrophil development, such as prostaglandin E_2_ (PGE_2_).^51^ Moreover, PGE_2_ prolongs neutrophil persistence by increasing the expression of antiapoptotic factor Nr4a2 and Nr4a3, and strengthens ROS production.^50,51^ The CXCL12-CXCR4 axis also promotes the retention of neutrophils in the lungs and inhibits their circulation.^52^ However, the existence of TRNs in other tissues is less defined.

In the tumor microenvironment (TME), the scRNA-seq of NSCLC tumor tissues identified two kinds of TRNs, including tumor-associated neutrophils (TANs) and normal adjacent tissue-associated neutrophils.^84^ TANs downregulate CXCR2 and SELL, the markers of mature neutrophils, whereas normal adjacent tissue-associated neutrophils highly express them.^84^ The common markers of TANs include OLR1, VEGFA, CXCR4, and CD83.^84^ These TANs are deeply involved in TME reshaping and regulation of cancer immunity.

Although studies focus on the regulatory mechanisms and biological significance of tissue-resident neutrophils are less, we think that investigating this neutrophil subpopulation will help us understand the role of neutrophils in diseases, especially infection and cancer.

### Tissue-resident mast cells

Mast cells are distributed in many organs, especially the respiratory tract and digestive tract where they are directly exposed to external environments.^344^ In rodent animal studies, tissue-resident mast cells can be divided into two subpopulations, connective tissue mast cells (CTMCs) and mucosal mast cells (MMCs).^345^

Embryonic CTMCs derive from three different hematopoietic progenitors in succession distributing into many tissues for maturation before birth.^26–28^ Postnatal CTMCs in most connective tissues mainly derive from late EMPs, whereas early EMPs specifically give rise to CTMCs in adipose tissue and pleural cavities.^20^ MMCs are mainly distributed in the epithelium of the respiratory tract and gastrointestinal tract. They mostly derive from definitive hematopoietic progenitors and adult cecum and utero MMCs can be replaced by bone marrow-derived HSCs.^20,37^

The adult skin-resident mast cells and utero-resident CTMCs minimally depend on bone marrow HSCs-derived cells for renewal.^33–37^ In contrast, bone marrow-derived mast cell progenitors partially get involved in the skin-resident mast cell pool and adaptively acquire CTMC phenotypes in the atopic dermatitis-like skin model.^42^ In addition, blood mast cell progenitors can also be recruited into the lung and gut to reside.^35,346,347^ However, the specific constitutions and the regulatory mechanisms of adult mast cells in different tissues are not confirmed. MMCs persistently reside in tissues for a relatively shorter life than CTMCs.^348–350^ A study explained that the Fcγ-induced cell apoptosis mechanism made MMCs more prone to apoptosis, seemingly resulting in the different tissue persistence of two mast cell populations.^351^ Especially, MMCs can migrate to infectious niches and expand.^352–355^ Therefore, we speculate that MMCs usually exist in tissues that are frequently exposed to allergens and pathogens, which also promote the short persistence of MMCs. However, the origin and development of human tissue-resident mast cells are unclear.

scRNA-seq analyses help people find more tissue-resident mast cell subtypes with high heterogeneity and plasticity in different tissues, such as skin, esophagus, trachea, and peritoneum.^87,135,144,345,356–361^ For example, skin-resident mast cells mainly upregulate genes involved in the development signaling pathway, including Lgr4, Smo, and Igf2r, and also TF SOX7.^87,349^ Mcpt1 and Mcpt2 are expressed in esophagus-resident mast cells.^349^ Lipf is enriched in tracheal-resident mast cells, and Itgb2 and Bmp2 are upregulated in peritoneal-resident mast cells.^349^ In addition, mast cells distributed in different tissues can produce different factors even during embryogenesis, such as granulocyte-macrophage-colony-stimulating factor (GM-CSF), vascular endothelial growth factor (VEGF), and platelet-derived growth factor (PDGF), which may contribute to specific tissue development.^87^

Tissue-resident mast cells can secrete cytokines, chemokines, and growth factors to regulate physiological or pathological processes, such as regulating angiogenesis by secreting VEGF, TGF-β, and CXCL18, and promoting vascular and blood-brain barrier permeability and facilitating fibrosis of chronic diseases dependent on TGFs and fibroblast growth factors.^362^ In addition, mast cells also mediate wound healing and nerve regulation.^363–365^ Although the role of mast cells in allergic inflammation is familiar, whether mast cells dominate other immune events such as its effects on cancer immunity is unclear.^363,366–368^

## Roles of TRICs in diseases

Given the wide existence and crucial functions of TRICs in tissues, the investigation of TRICs in pathological processes is important for deepening our understanding of pathogenesis and disease therapies. Here, we delineate the roles of TRICs in tissue homeostasis and repair, autoimmune diseases, infectious diseases, cancer, and others (Table 1).^369–373^

**Table 1:** Roles of TRICs in regulating different inflammatory diseases

Disease type	Specific disease	Cell type	Functions and mechanisms	Prognostic value	Ref.
Autoimmune disease	Psoriasis	CD8^+^TRM cell	Aggravate the skin inflammation by producing IL-17A	Negative	^416^
		CCD4^+^TRM cell	Aggravate the skin inflammation by producing IL-22	Negative	^417^
	Vitiligo	CD8^+^TRM cell	Aggravate the skin inflammation by producing TNF-α and IFN-γ	Negative	^420^
	RA	CD8^+^TRM cell	Promote joint injury and RA recurrence by recruiting circulating effector T cells	Negative	^393^
		Tissue-resident macrophages	Attenuate joint inflammation by constructing the intra-articular immunological barrier	Positive	^407^
	PsA	CD8^+^TRM cell	Promote joint injury and RA recurrence by producing IL-17A	Negative	^19^
	LN	CD8^+^TRM cell	Aggravate the renal inflammation by producing cytokines	Negative	^454^
		Tissue-resident macrophage	Promote kidney inflammation by recruiting monocytes, and producing cytokines that support the development of autoantibody-producing B cells	Negative	^456^
	ANCA-associated GN	CD8^+^TRM cell	Aggravate the renal inflammation by producing IL-17A	Negative	^106^
	MS	CD8^+^TRM cell	Aggravate the brain inflammation by producing cytokines and recruiting autoreactive T cells into the brain	Negative	^426^
		Microglia	Promote disease progression by producing proinflammatory cytokines or activating T cells	Negative	^447^
		Tissue-resident ILC3	Attenuate neuroinflammation by inducing immune tolerance and inhibiting autoimmune T cells	Positive	^433^
		Tissue-resident MAIT cell	Promote disease progression by producing IL-17	Negative	^434^
	SS	Tissue-resident macrophage	Promote disease progression by inducing proinflammatory T cell-mediated response	Negative	^457^
		CD8^+^TRM cell	Promote disease progression by producing TNF-α and IFN-γ	Negative	^97^
	Vasculitis	Tissue-resident macrophage	Promote disease development by inducing proinflammatory cell infiltration and cytokine production	Negative	^460^
	IBD	CD4^+^TRM cell	Aggregate the bowel inflammation by producing proinflammatory cytokines	Negative	^449^
		Tissue-resident MAIT cell	Aggravate the bowel inflammation by producing IL-17	Negative	^450^
	Autoimmune cholangitis	CD8^+^TRM cell	Promote disease development by inducing the apoptosis of small bile duct epithelial cells and exhibit enhanced cytotoxicity	Negative	^95^
	Autoimmune hepatitis	CD8^+^TRM cell	\	Negative	^451^
Infectious disease	Influenza virus	trNK cell	Make immune responses to infected cells by producing cytokines and cytotoxic molecules	Positive	^289^
		Tissue-resident macrophage	Defend against influenza infection by producing type I IFN	Positive	^466^
		Tissue-resident ILC1	Defend against influenza infection by producing IFN-γ	Positive	^484^
		Tissue-resident Vγ9Vδ2 T cell	Defend against influenza infection by producing IFN-γ	Positive	^489^
		CD8^+^TRM cell	Defend against influenza infection by producing IFN-γ, TNF-α, and cytotoxic molecules	Positive	^151^
		BRM cell	Defend against influenza infection by producing enhanced antibodies	Positive	^55^
	HSV	CD4^+^TRM cell	Defend against HSV by producing IFN-γ and IL-4	Positive	^116^
		Tissue-resident macrophage	Defend against HSV by producing proinflammatory cytokines	Positive	^494^
		CD4^+^TRM cell	Defend against HSV by producing IFN-γ	Positive	^502^
		CD8^+^TRM cell	Defend against influenza infection by producing IFN-γ, TNF-α, and cytotoxic molecules	Positive	^500^
	HIV	CD8^+^TRM cell	Defend against HIV by producing IFN-γ and CD107a	Positive	^510^
		CD8^+^TRM cell	Defend against HIV by enhancing local humoral immunity	Positive	^514^
		Tissue-resident macrophage	Aggravate HIV infection by supporting their persistence	Negative	^519^
	HBV	CD8^+^TRM cell	Defend against HBV by producing IFN-γ, TNF-α and IL-2	Positive	^162^
		Tissue-resident γδT cell	Defend against HBV by producing IFN-γ	Positive	^526^
	MTB	Tissue-resident macrophage	Defend against MTB by initiating the innate immune response	Positive	^538^
		Tissue-resident γδT cell	Defend against MTB by producing IL-17	Positive	^549^
		Tissue-resident ILC3	Defend against MTB by initiating the innate immune response	Positive	^551^
		CD4^+^TRM cell	Defend against MTB by producing IL-17	Positive	^547^
		Tissue-resident MAIT cell	Defend against MTB by producing IFN-γ and GZMB	Positive	^552^
	S. pneumoniae	Tissue-resident macrophage	Defend against S. pneumoniae by producing ROS and nitric oxide	Positive	^556^
		Tissue-resident macrophage	Reduce tissue inflammation by clearing apoptotic PMN during the S. pneumoniae infection	Positive	^558^
		Tissue-resident ILC3	Defend against S. pneumoniae by producing IL-17 and IL-22	Positive	^562^
		Tissue-resident γδT cell	Maintain tissue homeostasis during the resolution phase after infection	Positive	^561^
		BRM cell	Defend against S. pneumoniae by inducing humoral responses	Positive	^58^
		CD4^+^TRM cell	Defend against S. pneumoniae by producing IL-17	Positive	^565^
		CD8^+^TRM cell	Defend against S. pneumoniae by producing IFN-γ	Positive	^568^
	S.aureus	Tissue-resident macrophage	Defend against S.aureus by producing ROS and nitric oxide	Positive	^554^
		CD4^+^TRM cell	Defend against S.aureus by producing IL-17 and IFN-γ	Positive	^573^
	Malaria	CD8^+^TRM cell	Eliminate parasite-infected hepatocytes by producing IFN-γ, TNF-α and IL-2	Positive	^737^
	Leishmaniasis	CD4^+^TRM cell	Defend against parasites by producing IFN-γ and recruiting inflammatory monocytes and circulating T cells	Positive	^583^
		Tissue-resident macrophage	Produce anti-inflammatory cytokines and promote parasite growth	Negative	^589^
Transplantation	Lung transplant	CD8^+^TRM cell	\	Positive	^719^
		Tissue-resident macrophage	Produce inflammatory cytokines to activate immune responses against allograft	Negative	^719^
	Skin transplant	TRM cell	\	Negative	^721^
	Kidney transplant	CD8^+^TRM cell	\	Negative	^723^

*TRM* tissue-resident memory T, *BRM* tissue-resident memory B, *ILC* innate lymphoid T, *MAIT* mucosal-associated invariant T, *trNK* tissue-resident natural killer, *IFN-γ* interferon-γ, *TNF-α* tumor necrosis factor-α, *GZMB* granzyme B, *RA* rheumatoid arthritis, *PsA* psoriatic arthritis, *LN* lupus nephritis, *ANCA-associated GN* anti-neutrophil cytoplasmic antibody (ANCA)-associated glomerulonephritis (GN), *MS* multiple sclerosis, *SS* Sjögren′s syndrome, *IBD* immune bowel disease, *HSV* herpes simplex virus, *HIV* human immunodeficiency virus, *HBV* hepatitis B virus, *MTB* mycobacterium tuberculosis, *S. pneumoniae* Streptococcus pneumoniae, *S. aureus* Staphylococcus aureus, *PMN* polymorphonuclear neutrophil

### Tissue homeostasis and repair

Tissue-resident myeloid TRICs are the dominant regulators in tissue homeostasis, covering tissue repair, inflammatory damage inhibition, and stability of tissue architecture.

Tissue-specific macrophages have adaptive functions linked to their environmental backgrounds.^374^ They can resemble sensors and react to many environmental signals, such as pH, temperature, hypoxia, and stress, thus maintaining tissue homeostasis.^313^ In the process of tissue repair, macrophages can suppress neutrophil activation and inflammatory damage, and recruit myofibroblasts that contribute to tissue remodeling.^375–377^ For example, Kupffer cells participate in immune tolerance by suppressing CD4^+^ and CD8^+^T cell activation,^378,379^ triggering antigen-specific CD8^+^T cell apoptosis,^380^ and promoting the expansion of regulatory T cells (Tregs).^379^ In the gut, resident macrophages maintain intestinal functions and homeostasis. The depletion of this cell population will result in neuronal apoptosis and blood vessel leakage, decrease neuron secretion, and impair intestine motility.^168,327^ In addition, tissue-resident macrophages also participate in regulating homeostatic energy expenditure and heat generation by controlling sympathetic innervation in brown adipose tissues. Mecp2 deletion in adipose-resident macrophages will disrupt their cross-talks with neurons, resulting in decreased production of thermogenic factors by adipocytes, which are controlled by sympathetic nerve axons.^178^ Therefore, tissue-resident macrophages are multifunctional across different tissues.

Studies showed that tissue-resident mast cells regulated hair follicle growth, bone metabolism, and mucosal integrity in the intestine.^142,143^ The bone quality and strength of the mice with mast cell elimination were impaired.^175,176^ Intestine-resident mast cells contribute to maintaining mucosal barrier homeostasis by regulating epithelial cell activity and mucosal integrity.^144,145^ They also reverse tissue injury and promote wound healing.^381^ In wound sites, mast cells produce IL-4 and nerve growth factors to induce the proliferation and migration of fibroblasts to facilitate tissue healing.^133–135^

As for TRNs, some studies reported that they function in regulating vascular growth and repair in the lung and intestine, as well as hematopoiesis.^49,342,382,383^

Lymphoid TRICs are also involved in regulating tissue homeostasis, although few studies focus on this issue. An example is the regulatory feedback loop between ILC2s and stromal cells in white adipose tissues. ILC2s induce tissue-resident multipotent stromal cells to produce eotaxin and to recruit eosinophils for improving tissue homeostasis.^384^ Concurrently, white adipose tissue-resident mesenchymal stromal cells promote ILC2 to produce type 2 cytokines and similarly facilitate eosinophil recruiting.^385^ In addition, tissue-resident iNKT cells maintain adipose tissue homeostasis by inducing anti-inflammatory phenotypes of macrophages and suppressing Treg cell activation.^258^

### Autoimmune disease

**Fig. 4:**
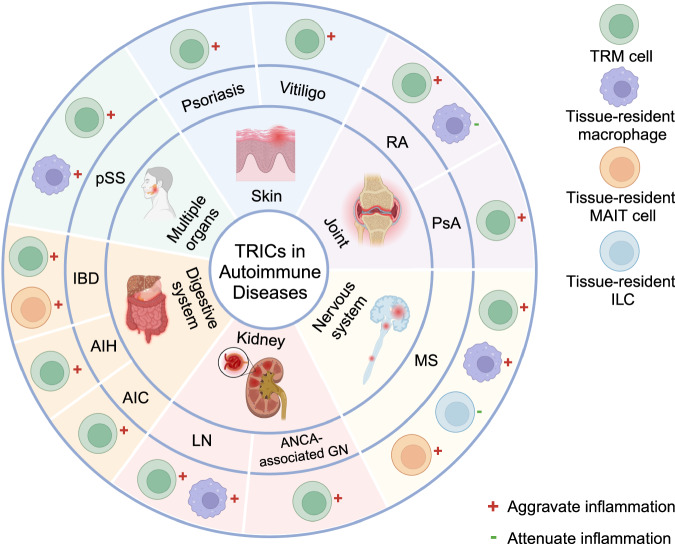
The regulatory roles of TRICs in autoimmune diseases. The red “+” means that the TRIC type mainly plays an inflammation-promoting role in corresponding autoimmune diseases, which will exacerbate disease progression. The green “-” means that the TRIC type can inhibit immune activity to attenuate diseases. RA rheumatoid arthritis, PsA psoriasis arthritis, MS multiple sclerosis, LN lupus nephritis, ANCN-associated GN anti-neutrophil cytoplasmic antibody-associated glomerulonephritis, AIC autoimmune cholangitis, AIH autoimmune hepatitis, IBD immune bowel disease, pSS primary Sjögren’s syndrome. This figure is created with BioRender.com

The overactivation of immune systems will cause exacerbated inflammation and extensive organ damage, finally leading to the initiation of autoimmune diseases.^386,387^ However, the pathogenic mechanisms of autoimmune diseases are not comprehensively studied. Many studies have investigated that TRICs are closely associated with the development of autoimmune diseases, and some of them will become potential therapeutic targets (Fig. 4).^8,388,389^

#### TRICs in autoimmune arthritis

Rheumatoid arthritis (RA) is an autoimmune disease with high incidences. The most common drug is disease-modifying antirheumatic drugs (DMARDs),^390,391^ though not all patients respond well to treatment. Therefore, it is important to explore new therapeutic targets.^392^ A better understanding of the pathological mechanisms of RA initiation and recurrence may help break through the barrier. Notably, the long-term tissue-resident properties of TRICs contribute to the pathology of RA.

CD8^+^T cells have been found to exist in the synovial fluid of RA patients for a long time. They exhibit canonical phenotypic of TRM cells, such as upregulating the expression of CD69, CXCR6, and CD49a, and downregulating S1PR1 and KLF2.^393,394^ In addition, These CD8^+^TRM cells prefer to uptake fatty acid and display enhanced activities in response to stimulation.^393^ The long-term resident synovial CD8^+^TRM cells can recruit circulating effector T cells by releasing CCL5 to aggravate joint injury and RA recurrence.^393,395,396^ Some studies also indicated that TRM cells may promote the recurrence of RA by perforin-mediated histone citrullination and the production of anti-citrullinated protein antibodies, which are the main characteristics of RA pathogenesis.^394,397–399^ Therefore, TRM cells may be the key regulator of RA recurrence, and their features are reversely influenced by the tissue environments of RA. CD8^+^T cells with similar phenotypes of TRM cells are also found in the tissues of psoriatic arthritis (PsA) patients, such as synovial fluid and the skin.^19,199,400,401^ However, their roles in regulating PsA are not confirmed.

The local composition of CD8^+^TRM cells in PsA and RA are distinct. The proinflammatory type 17-like CD103^+^CD69^+^CD8^+^TRM cells are specifically aggregated in the synovial fluid of PsA, whereas cytotoxic and Treg-like CD8^+^TRM cells are found in both RA and PsA.^19^ Whether the different phenotypes of TRM cells in the two arthritis attributed to the plasticity of TRM cells in different environments and the specific environmental stimulators needs further validation.

The roles of tissue-resident macrophages in RA have also been studied. In contrast to circulating monocyte-derived macrophages that mainly promote inflammation in RA,^402–404^ a recent study demonstrated that CX3CR1^+^ synovial lining-resident macrophages exhibited characteristics of epithelial cells. These tissue-resident macrophages limit joint inflammation by constructing a membrane-like immunological barrier in the synovial lining.^405–408^ Furthermore, after accessing the synovial niche, circulating monocytes can differentiate into tissue-resident macrophages, which mitigates the chronic inflammation of RA.^409^ These studies suggest the effector roles of tissue-resident macrophages and TRM cells in RA, but further studies on manipulating these TRICs to treat autoimmune arthritis are needed.

#### TRICs in skin-autoimmune diseases

Autoimmune skin diseases include psoriasis, vitiligo, et al. Many studies also revealed that TRIC subpopulations participate in the pathogenetic process of these diseases.^410^

Psoriasis is a chronic, relapsing autoimmune skin disease induced by IL-17A.^411–413^ The existence of TRM cells usually aggravates psoriasis. CD8^+^TRM cells have been identified to exacerbate skin inflammation in the psoriatic skin by producing IL-17A.^414–416^ Moreover, IL-22 produced by CD69^+^CD4^+^TRM cells also enhanced psoriatic inflammation by targeting epidermal keratinocytes, a predictive feature of psoriatic skin.^417,418^ Therefore, strategies for eliminating TRM cells may be effective in treating psoriasis.

Vitiligo is caused by the loss of melanocytes, and the main clinical manifestation includes recurrent depigmented patches.^419^ CD103^+^CD69^+^CD8^+^TRM cells with high levels of IFN-γ and TNF-α production have been identified in the skin lesions.^420,421^ These TRM cells synergize with circulating memory T cells to exacerbate the disease.^422^ Another study indicated that melanocyte-specific CD103^+^CD69^+^CD49a^+^CD8^+^TRM cells produced IFN-γ after recognizing Melan-A to perform cytotoxic killing.^415^ Interestingly, these CD8^+^TRM cells express CD122, a subunit of the IL-15 receptor, and anti-CD122 antibody treatment ameliorates the vitiligo lesion in the mice model.^18^ In addition, JAK inhibitors can reverse the depigmentation of vitiligo lesions due to the JAK/STAT signaling pathway being the driving pathway for cytokine production in TRM cells, which stresses that TRM cells are one of the feasible therapeutic targets of vitiligo.^421^

#### TRICs in nervous system-autoimmune diseases

Autoimmune diseases involving the nervous system are more harmful. The complicated pathogenesis and limited treatment strategies lead to unfavorable treatment outcomes and poor prognoses of patients.

The scRNA-seq studies showed that CD8^+^TRM cells, CD4^+^TRM cells, and trNK cells existed in the cerebrospinal fluid of MS patients.^423–425^ CD8^+^T cells infiltrating in the recurrent MS lesions display tissue-resident phenotypes, contributing to the compartmentalized inflammation in the brain.^426^ Mechanistically, TRM cells interact with other immune cells to develop their tissue-resident characteristics in the CNS. TGF-β released by Treg cells promotes the CD103 expression in the brain-infiltrated T cells.^427^ Moreover, PD-L1^+^glial cells combine with brain-CD103^+^CD69^+^CD8^+^TRM cells expressing PD-1, and promote their development.^428^ CD8^+^TRM cells can trigger pathological neuroinflammation and impair neurons with the assistance of CD4^+^T cells.^429,430^ Moreover, CD103^+^CD69^+^CD49a^+^CD8^+^TRM cells located in the white matter lesions of progressive MS have been demonstrated to promote inflammatory activity.^431^ Therefore, CD8^+^TRM cells are likely to be the pathogenic factors for MS progression.

T-bet-dependent NKp46^+^ILCs also get involved in driving CNS autoimmune disorders. They establish a proinflammatory environment for T helper type (Th)17 cells by secreting cytokines and matrix metalloproteinases, and all of these biological factors promote the migration of Th17 cells into CNS parenchyma.^432^ In contrast, tissue-resident ILC3s can suppress neuroinflammation by forming immune tolerance and eliminating autoimmune T cells.^433^ MAIT cells also promote MS progression by producing IL-17.^434^ The non-myeloablative autologous hematopoietic stem cell transplantation limited the proinflammatory process in MS by depleting IL-17-producing MAIT cells.^435^ These results suggest that IL-17-related lymphocytes are one of the drivers for CNS autoimmune diseases.

Microglia plays a dual role in nervous system-related autoimmune diseases.^436,437^ Regarding disease suppression, they can generate anti-inflammatory cytokines in favor of the differentiation of immunosuppressive cell populations such as Treg and Th2 cells, and display protective roles in neurological disorders.^438,439^ However, activated microglia will damage neurons in some cases. Microglia release harmful molecules such as reactive oxygen and nitrogen species and glutaminase,^440,441^, or secrete inflammatory molecules such as TNF-α.^442^ Moreover, they also promote the differentiation of pathogenic CD4^+^T cells, Th1, and Th17 cells which facilitate the progression of experimental autoimmune encephalomyelitis, a general model for studying MS.^443,444^ Blocking microglia alleviated the experimental autoimmune encephalomyelitis.^445,446^ Microglia also serve as APCs to present myelin antigens to activate T cells and then motivate the intense inflammatory responses.^447,448^ Therefore, microglia is recognized as the pathogenic participant in MS.

#### TRICs in digestive system-autoimmune diseases

TRICs also mediate the initiation and progression of several kinds of inflammatory bowel disease (IBD). CD103^+^CD69^+^CD4^+^TRM cells were found in the mucosa of patients, and the density of them was positively correlated with disease severity.^449^ Intriguingly, the deletion of Hobit and Blimp, the important TFs for the development of TRM cells, indeed induced IBD remission.^449^ Activated MAIT cells derived from IBD patients also exhibit high IL-17-secreting phenotypes, related to aggravating inflammation.^450^

Liver-CD8^+^TRM cells are found to induce the apoptosis of intrahepatic small bile duct epithelial cells and contribute to autoimmune cholangitis. Interestingly, they can express PD-1, and PD-1-targeted chimeric antigen receptor (CAR)-T therapy eliminated these CD8^+^TRM cells and achieved cholangitis remission.^95^ Scientists found the enrichment of TGF-β, IL-15, and E-cadherin in the liver of autoimmune hepatitis (AIH) patients, which orchestrated tissue residency of CD8^+^TRM cells.^451^ The infiltration levels of CD8^+^TRM cells are positively related to disease severity. Long-term tissue inflammation will lead to chronic organ fibrosis. MAIT cells have been found to promote liver fibrosis by producing IL-17A to activate hepatic stellate cells, an important component of liver fibrosis.^452^

#### TRICs in autoimmune nephritis

Lupus nephritis (LN) is a common complication of systemic lupus erythematosus.^453^ CD103^+^CD69^+^CD8^+^TRM cells are found to infiltrate the kidneys of LN patients or MRL/MpJ-*Fas*^*lpr*^/J (MRL/lpr) mice. These cells proliferate in situ to maintain themselves and elicit renal inflammation and flare.^454,455^ However, their disease-specific phenotypes are unclear. It was found that JAK/STAT signaling was important for the maintenance of renal TRM cells in MRL/lpr mice, and the application of tofacitinib, an inhibitor of JAK/STAT signaling, attenuated kidney inflammation.^455^ In addition, the depletion of kidney tissue-resident macrophages also inhibited inflammation by reducing the recruitment of monocytes and factors that encouraged localized B cells to produce autoantibodies.^456^

Moreover, the scRNA-seq profile of anti-neutrophil cytoplasmic antibody (ANCA)-associated glomerulonephritis (GN) showed higher infiltration levels of TRM17 cells than health.^106^ Genes associated with T cell activation, proliferation, and cytokine production were upregulated in the TRM cells derived from ANCA-associated GN.^106^ Therefore, CD8^+^TRM cells at least partially contribute to kidney autoimmune diseases.

#### TRICs in other autoimmune diseases

A study on primary Sjögren’s syndrome (pSS) indicated a cellular crosstalk dominated by TRICs to mediate autoimmune damage. CD11b^high^ tissue-resident macrophages secreted CCL22 to chemotaxis CD4^+^T cells with upregulated IFN-γ production, which aggravated local inflammation. Anti-CCL22 antibodies alleviated the progression of pSS.^457^ Similarly, the depletion of CD103^+^CD8^+^TRM cells by blocking CD103 is also helpful for SS inhibition.^97,458^ In contrast, salivary gland-trNK cells participate in attenuating SS progression. They may play protective roles in SS, fighting against the immune responses mediated by T cells.^459^

Cardiac tissue-resident macrophages are reported to promote autoimmune vasculitis by activating the CCL2/CCR2 axis, which can start the cascade of proinflammatory events as well as recruit circulating CCR2^+^monocytes and neutrophils.^460^

There remain some undetermined effects of TRICs in autoimmune diseases. For example, CD103^+^CD69^+^CD8^+^TRM cells have been identified as the predominant T cell population in the insulitic lesion of type 1 diabetes mellitus rather than classical cytotoxic CD8^+^T cells, which indicates the potentially important roles of TRM cells in diabetes mellitus.^461^ Studies on uterus-related autoimmune diseases showed that uterus-γδT cells upregulate IL-17 production after being activated, which may cause disease progression.^105,462,463^

Taken together, TRICs play inhibiting or promoting roles in different autoimmune diseases. However, limited to the research technologies and sample access, the available elucidation of TRICs in autoimmune diseases is still not enough to support clinical transformation.

#### Therapies targeting TRICs in autoimmune diseases

Repressing the excessive immune response is the most critical strategy for autoimmune disease treatment. We propose that the combination of routine immunosuppressive therapies with TRIC-targeting therapies may help amplify therapeutic efficacy.

First, blocking the critical molecules that contribute to the formation and maintenance of TRICs is feasible. For example, CD103 is the representative marker for most lymphoid TRICs, and a study found that CD103 inhibition remarkably alleviated the progression of SS.^97^ Fatty-acid-binding proteins 4 and 5 (FABP4/5) are indispensable for CD8^+^TRM cell survival, and their deletion also impair the formation of CD8^+^TRM cells.^464^

Second, therapeutic antibodies or small molecule inhibitors targeting effector cytokines or receptors of TRICs are implementable. Such as the anti-CD122 antibody achieves vitiligo remission.^18^ In the Lck-iDTR-engineered murine model with all T cells expressing DT receptors, synovial TRM cells are susceptible to DT-mediated depletion. The intra-articular injection of DT selectively depletes synovial TRM cells, resulting in attenuated joint flares in RA.^393^ In addition, IL-17A produced by CD8^+^TRM cells contributes to local inflammatory injury, and patients with PsA, autoimmune kidney disease, and psoriasis may benefit from IL-17-blockade therapy.^19,106,411,412^

Taken together, TRICs are potential candidates as disease-predictive biomarkers or therapeutic targets.

### Infectious disease

**Fig. 5:**
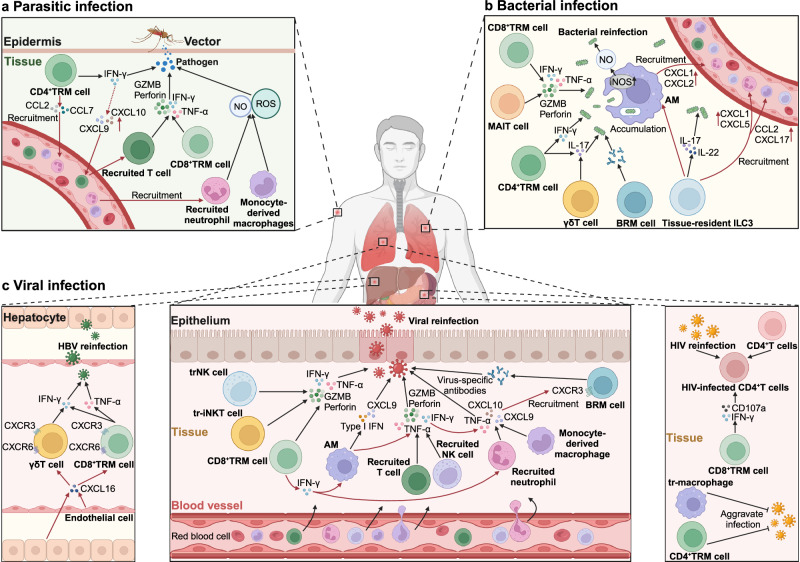
The anti-pathogen mechanisms of TRICs in infectious diseases. Upon pathogen reinfection, TRICs cooperatively perform immune defense functions. **a** After being stimulated by pathogens, CD4^+^TRM cells will rapidly produce IFN-γ, CCL2, and CCL7 to recruit circulating neutrophils and monocytes, which produce NO and ROS to defend against pathogens. CD8^+^TRM cells perform potent immune responses by producing IFN-γ and TNF-α. They also directly kill pathogens with perforin and GZMB. The high level of IFN-γ-inducible CXCL9 and CXCL10 in infected sites recruit circulating memory T cells to amplify local immune responses. **b** Upon bacterial reinfection, AMs and tissue-resident ILC3s quickly initiate the recruitment of circulating neutrophils and monocytes into the infected sites, while tissue-resident ILC3s further promote AM accumulation. Moreover, AMs enhance phagocytotic function and the autophagic clearance capacity by producing ROS, and they generate NO to directly kill bacteria. In addition, tissue-resident γδT cells, CD4^+^TRM cells, CD8^+^TRM cells, and MAIT cells also play important anti-bacterial roles by secreting regulatory cytokines or cytotoxic molecules. BRM cells can make rapid humoral immunity to resist bacteria reinfection. **c** Upon hepatitis virus infection, CXCL16 produced by hepatocytes and hepatic sinusoid epithelial cells promotes liver-resident CD8^+^TRM cells and γδT cells to retain in the liver. The two lymphoid TRICs exhibit potent immune responses against the HBV (Left). In the case of lung viral infections, multiple lung-TRICs cooperate to perform immune defenses. Lung-resident NK cells and iNKT cells highly produce IFN-γ, TNF-α, and cytotoxic molecules perforin and GZMB. Moreover, AMs produce chemokines to recruit circulating T cells and NK cells from the blood to enhance local immunity. AMs phagocytose virus-infected cells and defend against viruses via type I IFN. They also promote the recruitment of CXCR3^+^BRM cells by cooperating with neutrophils and monocyte-derived macrophages. After aggregating in infected sites, CXCR3^+^BRM cells differentiate into plasma cells to produce specific antibodies in response to virus infection. Moreover, TRM cells partially control the activation of innate cells in anti-viral protective immunity by producing IFN-γ (Middle). Upon HIV infection, CD8^+^TRM cells defend against HIV-infected CD4^+^T cells by producing IFN-γ and CD107a. In contrast, tissue-resident macrophages and CD4^+^TRM cells promote HIV survival and induce long-term infection (Right). AM alveolar macrophage, HBV hepatitis infection, HIV human immunodeficiency virus, IFN-γ interferon-γ, TNF-α tumor necrosis factor-α, GZMB granzyme B, IL interleukin, CCL C-C motif chemokine ligand, CXCL C-X-C motif chemokine ligand, NO nitrogen oxide, ROS reactive oxygen species, iNOS inducible nitric oxide sythase. This figure is created with BioRender.com

TRICs located in peripheral tissues are one of the major components of infection defense barriers. TRM cells, BRM cells, tissue-resident unconventional T cells, and trNK cells are induced after infection, and they keep long-term immune memory and generate potent immune responses against the reinfected pathogens. Mechanistically, activated TRICs will produce cytotoxic molecules to kill pathogens directly, or secrete cytokines and chemokines to recruit circulated immune cells to infected sites. Moreover, the primary localized myeloid TRICs also construct the defensive line at the environmental interface. Here, we provide an overview of TRICs in infectious diseases (Fig. 5).

#### Viral infection

Viruses are the most common pathogens that TRICs encounter. In this subsection, we introduce the roles of TRICs in several common or serious viral infections.

##### Influenza infection

Influenza infects the host through the respiratory tract where AMs are involved in establishing the first protective line. AMs initiate the infectious defense against the influenza virus by producing type I IFNs and recruiting circulating monocytes into infected sites.^465,466^ RNA-seq analysis also validated that AMs developed proinflammatory phenotypes during influenza infection.^107^ During the viral pneumonia resolution phase, the placenta-expressed transcript 1 generated by AMs aids in healing lung damage by promoting the regeneration of the lung epithelial barrier.^467^ However, chronic influenza infection also induces immune tolerance by damaging the migratory and phagocytotic capacities of AMs towards invasive bacteria, ultimately increasing the possibility of secondary bacterial infection.^107,468–470^ Researchers also detected a unique monocyte-derived AM population in mice with influenza infection. Although this cell population is distinct from resident AMs, they can offer prolonged protective immunity against bacterial infection by increasing IL-6 production.^471^ Therefore, the complicated lung-AMs pool significantly inhibits influenza infections.

The existence of virus-specific TRM cells in the lung after influenza infection has been validated in patient samples and animal models.^211,472–474^ TRM cells quickly expand and perform immune responses against invasive influenza by producing IFN-γ, TNF-α, and GZMB.^151,473,475^ Notably, influenza-specific CD8^+^TRM cells can offer cross-protection defense against various influenza virus strains, including influenza A, B, and C, which provides the basis for developing a broad-spectrum influenza virus vaccine.^475–478^ However, a study found that CD8^+^TRM cells aggregating in the aged lungs were dysfunctional and pathogenic. These CD8^+^TRM cells led to excessive inflammation and lung fibrosis.^479^ This finding suggests that CD8^+^TRM cells play bi-directional roles at different life stages.^480,481^ In addition, BRM cells are also induced by influenza stimulation in the lung. They differentiate into plasma cells and further perform humoral immunity upon virus rechallenge.^55,56,90^ Interestingly, innate and lymphoid TRICs can coordinate with each other to protect the host from influenza infection. For example, AMs promote BRM cells to migrate into secondary influenza-infected sites to enhance anti-virus immunity.^131^ Moreover, IL-18 produced by AMs contributes to the establishment of CD103^+^CD8^+^TRM cells and the expansion of CD8^+^TRM cells upon influenza reinfection.^482^ Conclusively, the development of vaccines that can induce TRM and BRM cells to amplify host defense against virus infection is a promising strategy.

Furthermore, other lymphoid TRICs also play important roles in protective immunity against influenza infection. First, lung-resident ILCs maintain lung homeostasis after influenza infection.^243,483^ The loss of lung-resident ILCs compromises the integrity of the lung epithelium barrier, but this can be reversed by replenishing the amphiregulin generated by lung-resident ILCs, a ligand for the epidermal growth factor receptor.^483^ Lung-ILC1s make rapid response upon influenza infection by producing IFN-γ at the early stage.^484^ Lung-ILC2s prolong the survival of influenza-infected mice by secreting IL-5 and further recruiting eosinophils at the late infectious stage.^485–487^ In addition, CD49^+^CD103^+^CD69^+^lung-trNK cells induced by the influenza A infection also enhance cytokine production targeting virus-infected cells in the lung.^154,289,488^ Tissue-resident γδT cells also eliminate influenza infection by producing IFN-γ.^489^ Therefore, lung-specific TRICs perform critical immune defense functions in controlling influenza infection. They are likely to be novel targets for influenza prevention and control.

##### HSV infection

HSV is the neurotropic herpesvirus that mainly invades the skin and mucosal tissues of the mouth, eyes, and reproductive tracts. HSV has two types, HSV-1 and HSV-2. The most common manifestation of HSV infection is mucocutaneous injuries, and some patients also have encephalitis.^490^ HSV-1 is the general leading cause of gingivostomatitis and keratoconjunctivitis, whereas HSV-2 preferentially leads to genital herpes.^491^ Mucosal immunity is the most critical defense against HSV infection. Given the wide distribution of TRICs in mucosal tissue sites, many studies focused on the protective roles of TRICs in HSV infection.

Microglia are involved in fighting HSV-1 infection.^492^ They resist HSV infections by producing proinflammatory cytokines like type I IFN and recruiting circulating monocytes into infected sites.^493,494^ Microglia deletion will aggravate herpes simplex encephalitis.^495^ However, microglia overactivation also impairs surrounding neurons and causes severe herpes simplex encephalitis.^496–499^ Hence, the moderate activation of microglia will have maximum inhibitory effects.^495^

TRM cells also respond to HSV infection rapidly.^500,501^ For example, CD4^+^T cells and CD8^+^T cells isolated from endocervical biopsy tissues of HSV-2 infected patients show tissue-resident phenotypes, and they defend against HSV by producing IL-4 and IFN-γ.^502^ In addition, people found CXCR3^+^CD8^+^TRM cells and CD69^+^CD14^+^CD8^+^TRM cells in recurrent ocular HSV-1 infection and vagina HSV-2 infection respectively.^116^ In the genital mucosa, CD69^+^CD103^−^CD4^+^TRM cells produce IFN-γ against HSV-2 infection.^116^ Therefore, virus vaccines promoting the formation of TRM cells in mucosal sites are significant for controlling HSV infection.

##### Human immunodeficiency virus (HIV) infection

HIV is the pathogen of acquired immunodeficiency syndrome (AIDS), which causes a huge burden of disease worldwide.^503^ Mucosal tissues are the primary barrier to preventing HIV invasion.^504^ HIV replicates in mucosal sites, and HIV-specific CD8^+^T cells are the main effector for controlling HIV infection.^505,506^ Many studies have validated the presence of HIV-specific CD8^+^TRM cells in the female genital tract,^507,508^ lymphoid tissues,^509^ gastrointestinal tract,^510,511^ and these cells can fight against HIV by performing polyfunctional responses, including producing IFN-γ, TNF-α, and other cytokines or chemokines, rather than enhancing cytotoxic capacity.^510,512,513^ In the mice model, the reactivated TRM cells can upregulate the local concentration of passively transferred anti-HIV antibodies in the genital mucosa.^514^ Except for TRM cells, in neurocognitive disorders caused by HIV infection, microglia potentiate neurotoxicity by raising glutaminase production, which in turn causes direct neuronal dysfunction and death.^440^

In addition, some subsets of TRICs are associated with persistent HIV infection. CD4^+^TRM cells and tissue-resident macrophages are the main reservoirs of persistent HIV infection, which support HIV replication and the progression toward AIDS.^515–519^ Adipose tissue has been recognized as the latent location for HIV infection.^520^ Staying in HIV-infected subcutaneous adipose tissues, Treg cells and PD-1^+^CD4^+^TRM cells construct an immunomodulatory milieu that protects HIV persistence.^521^

##### Hepatitis virus infection

Hepatitis virus is the pathogen of viral hepatitis, and it has six subtypes. The most common hepatitis virus subtypes include hepatitis A virus (HAV), HBV, and HCV.^522^ Different from HAV triggering acute liver inflammation, HBV and HCV can cause chronic liver inflammation which may progress to liver cirrhosis or even malignancies.^523^ Studies have found that liver-resident TRICs extensively participate in the pathogenic processes of chronic liver inflammation caused by hepatitis virus infection. The intrahepatic CD103^+^CD69^+^CD8^+^TRM cells can rapidly expand and produce a large amount of IFN-γ and TNF-α in response to HBV and HCV infection,^162,524,525^ Liver-resident γδT cells are also involved in HBV cleaning.^526^ However, whether their anti-virus activities harm normal liver parenchyma is yet to be determined.

Persistent HBV infection is an important contributor to hepatocellular carcinoma (HCC). Notably, HBV-specific CD8^+^TRM cells congregate in the livers of patients with HCC. These cells play cancer-inhibiting roles and are positively correlated with the patients’ relapse-free survival.^527,528^ Therefore, virus-specific CD8^+^TRM cells may serve as a predictive marker for HBV-induced HCC.

A recent study indicates that Kupffer cells can coordinate with liver-trNK cells to defend against pathogen infections.^529^ The two kinds of cells produce more potent immune responses to virus infection than bacterium infection, which is partially attributed to the different activation of toll-like receptor (TLR)-dependent signaling pathways in Kupffer cells and their regulatory effects on trNK cell cytotoxicity. Bacterial antigens combine with TLR2 or TLR4 expressed on Kupffer cells and transduced negative signals for trNK cells in an intracellular MyD88-dependent manner. In contrast, virus infection induces trNK cell activation through the MyD88-independent way.^529^ The roles of Kupffer cells and trNK cells in hepatitis virus infection are worth further exploring.

In addition to the above-mentioned types of infections, some studies also reported the role of TRICs in other viral infections. For example, CD8^+^TRM cells located in lung tissues rapidly secrete protective molecules such as IFN-γ and IL-2 after confronting with RSV, SARS-CoV-1, and SARS-CoV-2.^151,530^ In mice model with virus infection, brain-CD8^+^TRM cells provide immune defense against the reinfection of mouse polyomavirus,^531,532^ and tissue-resident ILC1s can make an early robust immune response against mouse cytomegalovirus in the liver.^484^ Generally, most TRICs contribute to protective immunity in response to various virus infections.

#### Bacterial infection

The genital tract, skin, and respiratory tract where TRICs are known to exist are the main entries for the exogenous bacterium to invade. Mucosal immunity serves as a crucial defense against bacterial infection.^533^ Especially, the pivotal roles that TRICs play in controlling bacterial infections within the respiratory tract serve as a representative example of TRICs’ function in bacterial infections, as outlined below.

##### Mycobacterium tuberculosis infection

Mycobacterium tuberculosis (MTB) is the pathogen of TB.^534^ AMs are the predominant immune cells to encounter MTB invasion, and they can phagocyte and kill intracellular pathogens in the early infectious stage by producing inducible nitric oxide synthase.^535–538^ However, MTB can escape the attack from innate immunity and reside in the niches enriched AMs which serve as a reservoir for MTB growth and help them to disseminate into the lung parenchyma.^537,539^ AM deletion has been identified to help inhibit MTB and enhance protective immunity in the lung.^540^

Numerous investigations have demonstrated the significant functions of antigen-specific lung-TRM cells in controlling MTB infection.^541,542^ Mycobacterium bovis Bacille-Calmette-Guérin (BCG) is the only prophylactic vaccine approved for treating TB.^543^ The administration of BCG can induce the formation of TRM cells that strengthen local anti-bacterial responses in the lung.^544,545^ CD4^+^TRM cells and CD8^+^TRM cells suppress MTB by secreting IFN-γ, TNF-α, and IL-2, and CD4^+^TRM cells also selectively generate IL-17 to further enhance the immune control.^546–548^ However, it is not clear whether long-term resident TRM cells contribute to excessive inflammation in the lung and their roles in the progression of chronic TB.

Other TRICs in the lung also contribute to the immune response against the MTB infection. For example, γδT cells provide a protective role against MTB infection by producing IL-17, which is important for the maturation of granulomas.^549,550^ Moreover, IL-17 and IL-22 produced by lung-resident ILC3s initiate early innate immune responses.^551^ CD103^+^CD69^+^MAIT cells aggregating in the tuberculous pleural effusions highly secrete IFN-γ and GZMB for MTB clearing.^552^ In summary, various lung-TRICs participate in regulating local immunity upon MTB infection.

##### Streptococcus pneumoniae (S. pneumoniae) infection

*S. pneumoniae* infection will cause pneumococcal pneumonia.^553^ The primary immunological line of defense against bacterial infection is innate immunity mediated by AMs. AMs can eliminate ingested *S. pneumoniae* by promoting phagolysosomal fusion and the production of proinflammatory cytokines, nitric oxide, and reactive oxygen species.^554–557^ Furthermore, AMs restrain local inflammation by eradicating apoptotic polymorphonuclear cells.^558^ During the resolution phase of infection, AMs also promote tissue repair, and lung-resident CD69^+^γδT cells prevent hyperinflammation by maintaining normal numbers of AMs and DCs.^559–561^

Lymphoid TRICs also play essential roles in resisting *S. pneumoniae* infection. The depletion of lung-BRM cells in mice suffering from pneumococcal pneumonia will enhance bacterial activities along with less antibody production.^58^ Additionally, ILC3s will undergo rapid expansion and produce IL-17 and IL-22 upon *S. pneumoniae* infection.^562–564^ As for TRM cells, studies found that intranasal administration of pneumococci induces CD4^+^TRM cells in local mucosa. These cells provide long-term protection against cognate antigen challenge.^565–568^ CD4^+^TRM cells and CD8^+^TRM cells upregulate cytotoxic factors and promote neutrophil infiltration, which positively amplifies the immune responses against pneumococci.^569,570^

##### Staphylococcus aureus (S. aureus) infection

AMs are the primary immune cells that defend against *S. aureus*.^554^ However, the impaired chemotaxis of AMs will lead to increasing infiltration of neutrophils upon *S. aureus* infection, thus breaking down the lung immune homeostasis and resulting in excessive inflammation.^107^ As to this problem, AMs can conceal the inhaled bacteria, which is useful for preventing the hyperinflammatory response induced by recruited neutrophils. In addition, lung-CD4^+^TRM cells inhibit *S. aureus* infection by producing IL-17 and IFN-γ.^571–573^ Consequently, vaccinations that stimulate CD4^+^TRM cells in the lung may help avoid *S. aureus* invasion.

#### Parasite infection

Given the complex symptoms and serious injury, parasite infections pose a significant threat to human life. Few investigations are examining the association between TRICs and parasite infection. Here we review the current understanding of TRICs in anti-parasitic immunity.

##### Malaria

Malaria is a dangerous parasite infection disease that greatly threatens human life, caused by plasmodium infection.^574^ During the primary infection, plasmodium will reproduce in the host liver. Then they enter into the blood and invade red blood cells.^575^ Therefore, eliminating malaria parasites in the liver is essential to halt malaria infection.

Liver-CD8^+^TRM cells elicited by the immunization with radiation-attenuated sporozoites will possess specific transcriptional profiles.^576^ Pathogen-specific CD8^+^TRM cells mediate the malaria-killing immune responses by generating IFN-γ, TNF-α, and IL-2 to destroy parasite-infected hepatocytes.^227,577^ Many studies are employing vaccines to treat malaria with the primary objective of eliciting malaria-specific liver-resident CD8^+^TRM cells, and the treatment outcomes are favorable.^578–581^

##### Leishmaniasis

Leishmaniasis is a zoonotic disease caused by the Leishmania spp. The common clinical manifestations include cutaneous, visceral, and mucocutaneous lesions.^582^ There are no specific vaccines for providing long-term protection from Leishmaniasis, so it is necessary to develop novel precautionary approaches.

Skin is the natural barrier for defending against parasite invasion. A study first demonstrates the presence of Leishmania-specific CD4^+^TRM cells in the infected skin. At the early infection stage, skin-CD4^+^TRM cells make rapid responses to the Leishmania parasite. This response is dependent on the recruitment of inflammatory monocytes rather than circulating memory T cells.^583,584^ CD4^+^TRM cells also produce IFN-γ and recruit circulating T cells into the skin lesion to carry out anti-parasite immune responses.^585,586^ Therefore, CD4^+^TRM cells are the candidate for developing vaccines against Leishmania.^587^

In contrast to parasite-specific CD4^+^TRM cells, skin-resident macrophages provide niches for parasite growth.^588^ After being stimulated by IL-4 secreted by local eosinophils, skin-resident macrophages produce CCL24 to recruit more eosinophils, which construct a positive feedback loop to promote cutaneous Leishmania spp infection.^589–591^

In addition to TRM cells and tissue-resident macrophages, tissue-resident mast cells also participate in regulating other parasite infections. For example, mast cells repel parasites in the intestine, such as worms and Toxoplasma gondii.^155–158^ The tryptase mouse mast cell protease-6 secreted by intestinal CTMCs attracts eosinophils to the chronic infection sites and significantly inhibits Trichinella spiralis invasion.^159^ The roles of other TRICs in regulating parasite infection are less reported.

Conclusively, certain TRICs are crucial for defending against pathogen infections, including viruses, bacteria, and some kinds of parasites. They become candidate targets for the prevention or treatment of these infectious diseases. It is waiting to explore whether TRICs could sustain potent and durable anti-infection activities throughout the infection cycle, and how to prevent them from over-activating.

#### Therapies targeting TRICs in infectious diseases

Based on the capability of TRM cells to provide long-term immune protection and their immune memory features, many researchers have developed vaccines targeting TRM cell activation.^592^ Studies found that vaccines can effectively induce TRM cells to defend against pathogens in infectious diseases.^234,581,593–596^ For example, mucosal immunization elicit HIV-specific CD8^+^TRM cells in the genital and respiratory tract.^593,597,598^ These cells perform potent anti-virus immune responses upon the HIV infection.^597^ In the lung, IL-17-producing CD4^+^TRM cells can be induced by vaccines to provide potent immune protection against MTB infection.^596^ Activated TRM cells also amplify the local immunity by activating and recruiting other immune cells into the infected mucosa.^593^

Notably, “Prime and pull” refers to a representative immunization strategy employing TRM cells.^596^ Firstly, a canonical parenteral vaccination is required to induce T cell responses, resulting in the existence of effector T cells in the circulation or infected sites. Subsequently, the “pull” step is the administration of chemokines or other stimulators to recruit activated T cells into organ-specific niches and develop tissue-resident features.^595^ This strategy has been successfully used in controlling HSV,^595,599,600^ influenza virus,^15^, and poxvirus infection.^16^ Of note, the delivery route of vaccines also influences the establishment of protective immunity, including T cell-mediated immune responses and antibody production.^592,596,601^ In contrast to intranasal and epidermal administration, intra-muscular vaccination cannot trigger robust T cell responses, possibly due to few dendritic cells in the muscles.^592,602^ Thus, the vaccination strategies need to be formulated according to the biological features of invasive pathogens.

### Cancer

#### Overview of TRICs in cancer

TME is a highly complex ecosystem where soluble mediators, ECM, and multiple cellular components assemble to establish the living environments for solid tumors. The cell components include many types of immune cells, stromal cells, and blood vessel-associated cells.^603,604^ As the first step of the cancer-immune cycle, tumor-derived neoantigens stimulate APCs to initiate the downstream immune responses. Different immune cells coordinate with each other to make cancer-killing or cancer-protective effects. In addition, the intercellular crosstalk not limited to immune cells also modulates cancer development, metastasis, therapy responses, and drug resistance.^605–607^ The long-term interaction between cancer cells and immune-killing effector components encourages cancer cells to acquire the ability to immune escape.^603^ Therefore, the persistent and sufficient cancer-killing abilities of immune cells are the decisive factor for cancer immunity. The long-term tissue residency properties render TRICs involved in the whole process of cancer immunity evolution.^608^

**Fig. 6:**
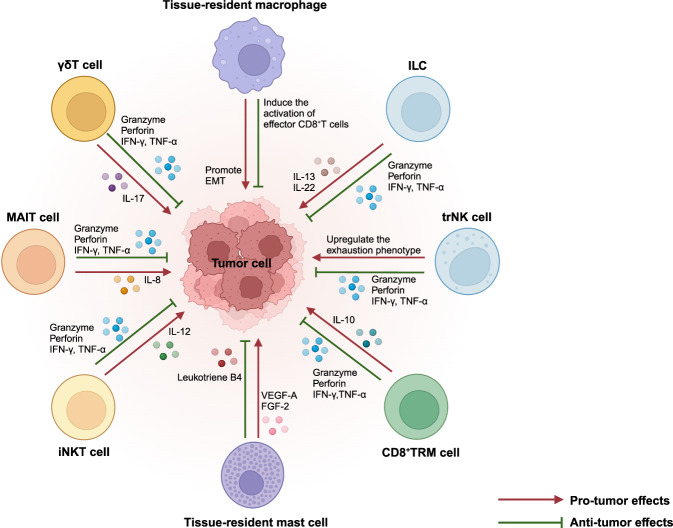
The roles of TRICs in cancer. Each TRIC shows opposite roles in tumor immunity. The red arrows indicate the pro-tumor effects, while the green arrows indicate the anti-tumor effects. VEGFA vascular endothelial growth factor-A, FGF-2 fibroblast growth factor 2. This figure is created with BioRender.com

As a non-negligible constituent of TIME, many studies have elucidated the role of TRICs in cancer immunity (Fig. 6, Table 2).^239,609–611^ Their phenotypes and their potential pro- or anti-tumor effects are regulated by the intrinsic mechanisms and external TME.^22^

**Table 2:** Dual regulation of different TRICs in cancer immunity

Cell name	Cancer type	Phenotypic marker	The impact on tumor	Mechanism	Clinical response	Prognostic value	Ref.
CD8^+^TRM cell	Melanoma	CD103, CD69, PD-1	Suppress tumor growth	CD8^+^TRM cells interplay with circulating memory T cells and amplify the anti-tumor immune response	\	Positive	^239^
		CD103, CD69, CD8A	Suppress tumor growth and maintain the immune equilibrium	CD8^+^TRM cells produce IFN-γ, TNF-α, and secret GZMB and perforin	\	Positive	^613^
	NSCLC	CD103, PD-1, TIM-3, CTLA-4	Kill tumor cells and magnify the cytotoxic anti-tumor immune responses	CD8^+^TRM cells promote the intra-tumoral CTL responses and increase the intraepithelial lymphocyte infiltration	The high density of these TRM cells is positively correlated with patient prognosis (DFS and OS)	Positive	^240^
		CD103, CD69, CD38, CD39, CD107a, CXCR6, PD-1, CTLA-4, TIM-3	Kill tumor cells and magnify the cytotoxic anti-tumor immune responses	CD8^+^TRM cells upregulate the expression of CD107a and produce IFN-γ, TNF-α, and secret GZMB and perforin.	The high density of these TRM cells is positively correlated with better long-term survival outcomes for patients	Positive	^197^
	BC	CD103, CD69, TIM-3, PD-1, CTLA-4, LAG-3, TIGIT	Kill tumor cells and maintain immunosurveillance	CD8^+^TRM cells produce IFN-γ and TNF-α	The gene signatures of TRM cells are positively correlated with improved patient survival	Positive	^738^
	Luminal-like BC	CD39, CD69, CD103, HLA-DR, TIGIT	Enhance degranulation capacity and magnify the cytotoxic anti-tumor immune responses	CD8^+^TRM cells produce IFN-γ and TNF-α	The expression of CD103 and/or CD39 of TRM cells is positively correlated with better prognosis (OS)	Positive	^633^
	TNBC	CD103, CD69, TIM-3, PD-1, CTLA-4, LAG-3	Kill tumor cells and make responses to ICB therapies (anti-PD-1 and/or anti-CTLA-4 therapy)	The number of CD8^+^TRM cells increases, and they produce IFN-γ and TNF-α	\	Positive	^241^
	HNSCC	CD103, CD39, CTLA-4, PD-1, TIGIT, TNFRSF9	Kill tumor cells and magnify the cytotoxic anti-tumor immune responses	CD8^+^TRM cells produce IFN-γ	The percentage of TRM cells is positively correlated with patient survival (OS)	Positive	^163^
	HGSC	CD103, CD69, PD-1, HLA-DR, Ki67, TIM-3, LAG-3, CTLA-4	Suppress tumor growth and magnify the cytotoxic anti-tumor immune responses	\	The existence of TRM cells in patients is correlated with increased HGSC-specific survival	Positive	^739^
	Cervical cancer	CD103, CD69, HLA-DR, PD-1, CTLA-4	Promote the identification of intraepithelial CD8^+^TIL in cervical cancer and serve as a biomarker for HPV16 E6/E7 targeted therapy	\	The infiltration level of TRM cells is positively correlated with patient prognosis (DSS and DFS)	Positive	^9^
	cSCC	CD103, CD39, CTLA-4, PD-1	Promote tumor metastasis and deactivate anti-tumor immune response	CD8^+^TRM cells upregulate the expression of immunosuppressive molecules (PD-1 and CTLA-4) and produce IL-10	The frequency of CD8^+^TRM cells is negatively correlated with patient survival (5-year DSS)	Negative	^205^
	HCC	CD103, CD39, PD-1, CTLA-4, TIM-3, LAG-3, TIGIT	Kill tumor cells and magnify the cytotoxic anti-tumor immune responses	The number of CD8^+^TRM cells increases, and they produce IFN-γ and TNF-α	The high density of these TRM cells is positively correlated with patient prognosis (OS)	Positive	^614^
	ICC	CD103, CD39, PD-1, CTLA-4, TIM-3, LAG-3, TIGIT	Kill tumor cells and magnify the cytotoxic anti-tumor immune responses	The number of CD8^+^TRM cells increases, and they produce IFN-γ and TNF-α	The high density of these TRM cells is negatively correlated with the advanced pathological stage	Positive	^614^
		CD103, CD69, CD39, CD38, HLA-DR	Magnify the cytotoxic anti-tumor immune responses and make responses to ICB therapies (anti-PD-1 therapy)	The number of CD8^+^TRM cells increases, and they produce IFN-γ and TNF-α	\	Positive	^615^
	ccRCC	CD103	Deactivate anti-tumor immune response	\	The high density of these TRM cells is negatively correlated with patient prognosis (OS)	Negative	^627^
Tissue-resident Vδ1^+^γδT cell	TNBC	CD69, CD107a, NKG2D	Magnify the cytotoxic anti-tumor immune responses and promote tumor remission	Vδ1^+^γδT cells produce IFN-γ and TNF-α	The high density of these Vδ1 + γδ cells is positively correlated with patient prognosis (PFS and OS)	Positive	^620^
	NSCLC	CD103, CD69, CXCR6, NKG2D	Kill tumor cells and maintain immunosurveillance	Vδ1^+^γδT cells produce IFN-γ, TNF-α, and secret GZMB and perforin	The high density of these Vδ1^+^γδ cells is positively correlated with patient prognosis (RFS)	Positive	^109^
	CRC	CD69, CD57, CD56, NKG2D	Suppress tumor progression and metastasis	Vδ1^+^γδT cells produce IFN-γ, TNF, and X-C motif chemokine ligand 2	The high density of these Vδ1^+^γδ cells is positively correlated with patient prognosis (OS)	Positive	^622^
Tissue-resident Vγ9Vδ2 TRM cell	HCC	CD103, CD69, CD49a, CXCR3, CXCR6	Kill tumor cells and magnify the cytotoxic anti-tumor immune responses	Vγ9Vδ2 TRM cells produce IFN-γ, IL-2 and secret GZMB	\	Positive	^621^
Tissue-resident iNKT cell	CRC	CXCR3, NK1.1	Kill tumor cells and magnify the cytotoxic anti-tumor immune responses	iNKT cells produce IFN-γ, TNF-α, and secret GZMB and perforin	\	Positive	^623^
Tissue-resident iNKT17 cell	Metastatic liver cancer	CD45	Promote tumor metastasis	IL-22 induced by iNKT17 acts on endothelial cells, which increases their permeability and promotes the cancer cell extravasation into the liver parenchyma	\	Negative	^609^
Tissue-resident MAIT cell	HCC	CD95, PD-1, CTLA-4, TIM-3	Promote tumor progression	MAIT cells reduce the production of Th1 cytokines and cytotoxic molecules, but they upregulate the production of tumor-promoting cytokines and the expression of inhibitory molecules	The high density of these MAIT cells is negatively correlated with patient prognosis (OS and RFS)	Negative	^629^
		FOXP3, CXCR3	Promote tumor progression	MAIT cells reduce the production of Th1 cytokines and express characteristics related to Tregs	\	Negative	^628^
	Melanoma	CD69	Promote tumor metastasis	MAIT cells suppress the activation of NK cells	\	Negative	^740^
	CCA	CD69, DNAM-1	Suppress tumor growth and metastasis	Activated MAIT cells enhance the anti-tumor function of NK cells	\	Positive	^624^
trNK cell	Lung cancer	CD27, CD94, CXCR3	Suppress tumor growth	trNK cells control tumor growth in an IFN-γ ‐dependent manner	\	Positive	^625^
	HCC	CD49a, PD-1, CD96, TIGIT, CXCR6	Promote the development of HCC	\	The percentage of trNK cells is negatively correlated with patient survival (OS and DFS)	Negative	^112^
	Ovarian cancer	CD49a, NKG2A	Exert anti-tumor activity	\	\	Positive	^626^
Tissue-resident ILC1	BC	CD49a, CD103	Suppress tumor growth and enhance cancer immunosurveillance	Resident ILC1s produce IFN-γ, TNF-α, and secret granzyme B	\	Positive	^638^
Tissue-resident macrophages	NSCLC	CD169, MARCO, HLA-DR, CD43	Promote tumor growth and invasion	Tissue-resident macrophages promote the EMT and facilitate the suppressive immune response	\	Negative	^610^
	Ovarian cancer	CD163, TIM4	Promote tumor progression and metastasis	Tissue-resident macrophages promote the EMT	\	Negative	^675^
	BC	FOLR2, HLA-DR, CD14	Magnify the cytotoxic anti-tumor immune responses	Tissue-resident macrophages interact with CD8^+^T cells through the PD-1 signaling pathway	The high density of tissue-resident macrophages is positively correlated with patient prognosis (OS)	Positive	^372^
	PDAC	CX3R1, CXCR4, CD64, CD115, PD-1, PD-L1	Promote tumor progression and pancreas fibrosis	\	\	Negative	^741^
	Metastatic liver cancer	TIM4, CLEC4F	Exert anti-tumor activity	Kupffer cells phagocyte tumor cells and recruit effector lymphocytes by producing various chemokines and cytokines	\	Positive	^44^

*NSCLC* non-small cell lung cancer, *PD-1* programmed cell death-1, *TIM-3* T cell immunoglobulin domain and mucin domain-3, *CTLA-4* cytotoxic T lymphocyte-associated antigen-4, *CTL* cytotoxic T lymphocyte, *DFS* disease-free survival, *OS* overall survival, *BC* breast cancer, *LAG-3* lymphocyte-activation gene-3, *TIGIT* T cell immunoglobulin and ITIM domain, *ICB* immune checkpoint blockade, *TNBC* triple-negative breast cancer, *HNSCC* head and neck squamous cell carcinoma, *HGSC* high-grade serous ovarian cancer, *TIL* tumor-infiltrating lymphocyte, *DSS* disease-free survival, *cSCC* cutaneous squamous cell carcinoma, *HCC* hepatocellular carcinoma, *ICC* intrahepatic cholangiocarcinoma, *ccRCC* clear cell renal carcinoma, *PFS* progression-free survival, *RFS* recurrence-free survival, *CRC* colorectal cancer, *iNKT* invariant natural killer T, *CCA* cervical cancer, *trNK* tissue-resident natural killer, *ILC* innate lymphoid cell, *EMT* epithelial-mesenchymal transition, *PDAC* pancreatic ductal adenocarcinoma, *TNFRSR* TNF receptor superfamily, *NKG* natural killer group, *DNAM-1* DNAX accessory molecule 1, *HLA* human leukocyte antigen, *FOXP3* Forkhead box protein P3, *MARCO* macrophage receptor with collagenous structure, *FOLR2* folate receptor beta, *TIM4* T cell immunoglobulin domain and mucin domain protein 4, *CLEC4F* C-type lectin domain family 4 member F

The lymphoid TRICs are the primary players involved in anti-tumor immunity. CD8^+^TRM cells have been reported to enhance cancer-killing responses in BC,^241,612^ melanoma,^239,613^ NSCLC,^197,240^ cervical cancer,^9^ high-grade serous ovarian cancer (HGSC),^163^ HCC,^614^ and intrahepatic cholangiocarcinoma.^614,615^ The levels of CD8^+^TRM cells have positive clinical prognostic value for these cancer patients. Mechanistically, CD8^+^TRM cells release TNF-α, IFN-γ, perforin, and GZMB to perform cancer-inhibiting functions.^239,613–615^ Interestingly, these tissue-resident molecules such as CD103 extensively expressed by TRICs also regulate cancer immunity, not limited to the residency-related effects. CD103 serves as a co-stimulator for TCR-mediated cancer-killing signals.^616^ In addition, TGF-β, IL-33, and IFN-γ could induce the formation of CD8^+^TRM cells.^219^ TGF-β promotes CD8^+^TRM cells to express CD103 and enhances their adhesion to E-cadherin on epithelial cancer cells.^193,617,618^ Mechanically, the recruitment and phosphorylation of ILK are induced by TGF-β binding to TFGBR. ILK then interacts with the CD103 intracellular domain and active PKB/AKT to transduce transmembrane integrin signals. This process strengthens the recruitment of CD103^+^CD8^+^tumor-infiltrating lymphocytes (TILs) into epithelial tumor sites and amplifies their anti-tumor responses.^619^

Innate-like lymphocytes are not restricted to specific antigen-receptor recognition and activation, so they can exert rapid responses. Especially, most of them have tissue residency characteristics.^120^ Some studies have reported that MHC-independent γδT cells secrete high levels of cytotoxic factors such as IFN-γ, GZMB, and TNF-α in TME to motivate anti-cancer activity in triple-negative breast cancer (TNBC),^620,621^ NSCLC,^109^ colorectal cancer (CRC),^622^ and HCC.^621^ The presence of γδT cells has been identified as a favorable prognostic marker.^109,620,622^ Similarly, iNKT cells display robust killing effects on CRC tumor cells, and MAIT cells also play a cancer-repressive role in cholangiocarcinoma (CCA).^623,624^ In studies of lung cancer and ovarian cancer, trNK cells are the main anti-tumor force. They can suppress tumor growth and metastasis by secreting proinflammatory cytokines and cytotoxic molecules.^625,626^

Moreover, myeloid TRICs also participate in regulating tumor immunity. A study showed that tissue-resident macrophages got involved in magnifying anti-tumor immune response through interacting with CD8^+^T cells, and their high infiltration correlated with improved overall survival in BC patients.^372^

However, TRICs play dichotomous roles in different cancer types, regarded as cancer-killing effector cells in some studies, yet showing cancer-protective activity in others. For example, in cSCC and clear cell renal carcinoma, CD8^+^TRM cells repress cancer immunity through upregulating inhibitory receptors such as PD-1 and CTLA-4, or secreting inhibitory cytokines, and their numbers are negatively correlated to patient prognosis and clinical survival.^205,627^ trNK cells suppress tumor growth and metastasis in lung cancer,^625^ but in a study of HCC, CD49a^+^liver-trNK cells expressing inhibitory molecules PD-1 and LAG-3 were associated with tumor progression.^112^ Moreover, MAIT cells inhibit anti-cancer immunity and promote tumor progression in HCC and melanoma, correlated to the shorter overall survival and recurrence-free survival of patients.^628,629^

Taken together, the regulatory effects of TRICs provide new insight into cancer immunity. However, the current exploration of TRICs is mainly based on animal studies. Breaking through the technical limitations of human research and verifying the reliability of animal research conclusions in humans is critical for future TRIC investigations.

#### Roles of lymphoid TRICs in regulating cancer immunity

T cells are the main force of anti-tumor immunity. Circulating CD8^+^T cells migrate into TME and perform cancer-killing effects upon stimulation by tumor antigens presented on DCs. However, it is hard for effector T cells to maintain long-term immune protection due to functional exhaustion or cancer-immune escape.^630^ Many studies found CD8^+^TILs aggregating in the TME acquired the classic phenotypic and transcriptional markers of TRM cells, including CD103 and CD69, which were indispensable for the long-term tissue residency of CD8^+^T cells in tumors.^631^ Anusha-Preethi Ganesan et al. discovered the isolated tumor-infiltrating CTLs from lung cancer patients having transcriptional profiles of CD8^+^TRM cells, and CD103^+^CD8^+^TRM cells were a favorable prognostic maker of lung cancer patients.^197^ Moreover, CD103^+^CD8^+^TRM cells have been identified in other epithelial tumors, including high-grade ovarian cancer and CRC.^632,633^

Mechanistically, TRM cells eliminate cancer cells through TCR-mediated cytotoxic effects. The functional initiation of TRM cells requires the activation of co-stimulating TCR signals, including E-cadherin-CD103, extracellular signal-regulated kinase1/2-phospholipaseCγ1, phosphoinositide-3-kinase-AKT, proline-rich tyrosine kinase 2, and paxillin (Pxn).^228,634,635^ Activated TRM cells produce cytotoxic molecules, including GZMB, perforin, and IFN-γ, and upregulate 4-1BB, CD27, and Ki67 expression, sustaining anti-tumor activities.^197,636^ Many cytokines engage in regulating the immune abilities of TRM cells. IL-15 promotes the establishment of TRM cells and ILCs in BC.^637,638^ In lung cancer, the interaction between CD103 and E-cadherin induces CCR5 recruitment at the immunologic synapse to further retain CD103^+^TILs.^639^

Lymphoid TRICs also directly regulate tumor progression in a cytokine-dependent manner. For example, IL-22 produced by tissue-resident iNKT cells or CD4^+^TRM cells promotes cancer metastasis at different stages. At the early stage of cancer metastasis, IL-22 produced by tissue-resident iNKT17 cells facilitates the extravasation of cancer cells into the liver, and CD4^+^TRM cell-derived IL-22 promotes tumor metastasis at the late stage. IL-22 neutralization can halt this process.^609,640^

In contrast, many studies have demonstrated that TRM cells play opposite roles in regulating anti-tumor immunity. TRM cells can accelerate cancer progression by secreting immune-repressive molecules or aiding tumor immune escape processes. In cSCC, the CD103^+^CD39^+^CD8^+^TRM cells promote tumor metastasis and inhibit anti-tumor response by increasing IL-10 production and upregulating the expression of exhaustion markers like PD-1 and CTLA-4.^205^ A study revealed that the pre-existing TRM-like cells in lung tissues strengthened the recruitment and activation of host T cells within TME, and they also expressed high PD-1 and IFN-γ.^641^ In addition, the pre-existing CD8^+^TRM cells induced by cancer vaccines facilitate anti-tumor immunity in head and neck tumors.^229^

The tertiary lymphoid structures (TLSs) are the aggregation of immune cells resembling SLOs anatomically present in non-lymphoid tissues upon chronic inflammation, including autoimmune diseases, infection, and cancer.^642^ TLS is mainly composed of a T cell-rich zone adjacent to a B cell follicle having germinal center characteristics and is surrounded by mature DCs. The existence of TLSs in cancer loci is always positively associated with the prognosis and clinical outcomes of patients.^643^ Many studies suggested that TLS formation is closely related to TRM cell evolution.^643,644^ In GC patients, CD103^+^CD8^+^TRM cells were located around TLSs in the cancerous areas, and the high enrichment of CD103^+^CD8^+^TRM cells in TLSs was correlated with favorable patients’ responses to anti-PD-1 therapy and prognosis.^645^ CD103^+^TRM cells were also set within TLSs in lung adenocarcinoma neoplastic tissues, and their high density was positively associated with TLS maturity.^646^ CXCL13 produced by CD103^+^TRM cells promotes the establishment of TLS.^647–649^ Therefore, we speculate whether some driver factors contribute to the coordinative formation of TLS and TRM cells to maintain long-term anti-tumor function.

Unconventional T cells also play important roles in regulating tumor progression. In TME, iNKT cells recognize cancer cells or others expressing CD1d-lipid antigen and clean them by secreting cytotoxic molecules.^259^ They also activate other effector immune cells, such as DCs and CD8^+^T cells, and simultaneously repress immunosuppressive cells to amplify the immune response.^110,267^ In pancreatic cancer liver metastasis and chronic lymphocytic leukemia model, iNKT cells reverse immunosuppressive TME by promoting the infiltration of effector T cells or restraining TAMs.^650,651^ Nonetheless, iNKT cells showed cancer-protective roles in some cancers.^609^

γδT cells can directly kill cancer cells by antibody-dependent cell-mediated cytotoxicity or induce other immune cells to amplify anti-tumor responses cooperatively in TME.^621,652^ Studies also found that CD103^+^ or CD69^+^tissue-resident Vδ1^+^γδT cells were significantly related to the improved clinical outcome of cancer patients.^109,620,622^ Especially due to the non-MHC restriction and powerful cytotoxic functions of γδT cells, a substantial number of cancer therapy developments focus on allogeneic γδT cells, including endogenous γδT cell activation.^653,654^ Tissue-resident γδT cells are also potential therapeutic targets to offer new avenues for cancer immunotherapy.

MAIT cells exist in primary and metastatic tumors and mainly perform immunosuppressive functions in cancers, including promoting angiogenesis, suppressing T cells and/or NK cells, producing immunosuppressive cytokines, and recruiting cancer-protective immune cells into TME, consequently promoting tumor progression.^624,628,655–658^ For example, MAIT cells accelerate HCC progression by reducing Th1 cytokines, producing immunosuppressive cytokines, and upregulating immune checkpoint PD-1 and CTLA-4.^628,629^ On the contrary, MAIT cells also strengthen immune surveillance by producing cytotoxic molecules in some tumors.^659^

ILCs also get involved in cancer immunity, and their phenotypes will be reshaped within TME.^660,661^ In the MMTV-PyMT mammary tumor model, IL-15 promotes the establishment of a tissue-resident ILC1s subgroup termed type 1-like ILCs, which exhibit cytotoxic functions against cancer cells by producing GZMB, IFN-γ, and TNF-α.^638^ In contrast, IL-13 produced by ILC2s is correlated with poor prognosis of cancer patients.^278^ ILC3s reshape the TIME of intestine cancers, and the IL-22 secreted by them promotes tumor development,^662^ suggesting that ILC2 and ILC3 cells play immunosuppressive roles.

Notably, NK cells would transform a lot after entering solid tumor sites. They upregulate the expression of CD103, CD49a, and some inhibitory receptors, along with impaired cancer-killing effects.^663^ The cancer-specific phenotypes of NK cells are caused by cytokines within TME, like TGF-β, PGE-2, and IL-15.^663^ The roles of trNK cells in cancer immunity are seemingly dichotomous, but the specific mechanisms are unclear. In liver cancer, NK cells have been found to accelerate cancer progression by upregulating immunosuppressive molecules, including CD96, PD-1, and TIGIT, and are identified as a poor prognostic factor.^112^ In contrast, trNK cells have higher degranulation levels for cytotoxic molecules in ovarian cancer and NSCLC to promote anti-cancer ability.^626,664^ Considering the advances in NK cell therapies such as CAR-NK therapy, it is essential to further elucidate the role of trNK cells in cancer.

#### Roles of myeloid TRICs in regulating cancer immunity

TAMs infiltrated in the TME are one of the important components in regulating tumor immunity, and they can interact with TRM cells to regulate their maintenance.^665,666^ In terms of TME, a study found that in early lung cancer, M1 type-TAMs enhanced the infiltration and maintenance of CD8^+^TRM cells in tumor sites by producing CXCL9 and fatty acids, which CD8^+^TRM cells depend on for survival and functions.^667^ In addition, another study also indicated that IL-15 secreted by TAMs contributed to the persistent residency of TRM cells in BC.^637^

Other tissue-resident macrophages regulate cancer processes by specific mechanisms. Kupffer cells play important roles in preventing tumor cell engraftment and restricting metastatic tumor outgrowth in the liver.^44,668–670^ They exert anti-tumor immune responses by phagocyting tumor cells and releasing various chemokines and cytokines to recruit effector T cells and NK cells into the tumor border.^44,45^ Thus, Kupffer cells can serve as promising targets for treating liver metastatic malignancies. Furthermore, FOLR2^+^tissue-resident macrophages have been identified in BC for their promoting effects on CD8^+^T cell infiltration.^372^ In CRC, scientists identified that FCN1^+^monocyte-like cells enriched in tumor tissues are the precursors of many tissue-resident macrophages, which finally develop into C1QC^+^ and SPP1^+^TAMs.^326^ C1QC^+^TAMs are highly related to IL1B^+^tissue-resident macrophage, and they perform cellular phagocytosis and antigen presentation. SPP1^+^TAMs mainly connect to NLRP3^+^tissue-resident macrophages, which interact with tumor-associated fibroblasts to promote tumor angiogenesis and tumor metastasis.^326^ Moreover, the LYVE1^+^macrophages and NLRP3^+^ macrophages are found in adjacent non-cancer tissues across many cancer types and have tissue-resident characteristics.^325^ LYVE1^+^tissue-resident macrophages contribute to constraining tissue inflammation in CRC, while NLPR3^+^macrophages appear to have proinflammatory effects.^311,326^

Notably, tissue-resident macrophages are also involved in the epithelial-mesenchymal transition (EMT) of cancer cells. EMT is a prerequisite process of epithelial tumor metastasis, describing the process of epithelial cells gradually changing into mesenchymal-like cells while also losing their original characteristics.^671–673^ Tissue-resident macrophages can accelerate tumor progression by regulating the EMT, such as AMs accelerating NSCLC progression by activating the EMT process.^610,674^ Similarly, CD163^+^TIM4^+^ omental macrophages promote ovarian cancer metastasis by supporting the EMT and the formation of cancer stem cells.^675^ In pancreatic ductal adenocarcinoma, tissue-resident macrophages evolved a profibrotic transcriptional profile. Eliminating these cells reduces tumor burden.^676^ However, the roles of TRNs and tissue-resident mast cells in cancer processes have not been reported.

Taken together, the tissue-residence features facilitate their long-term exposure to cancer cells and functions of regulating cancer immunity, which suggests the strategies motivating their immune activities are likely to achieve long-acting therapeutic efficacy. Further explorations are warranted to elucidate the aspects of cancer immunity dominated by TRICs.

#### Therapies targeting TRICs in cancer

**Fig. 7:**
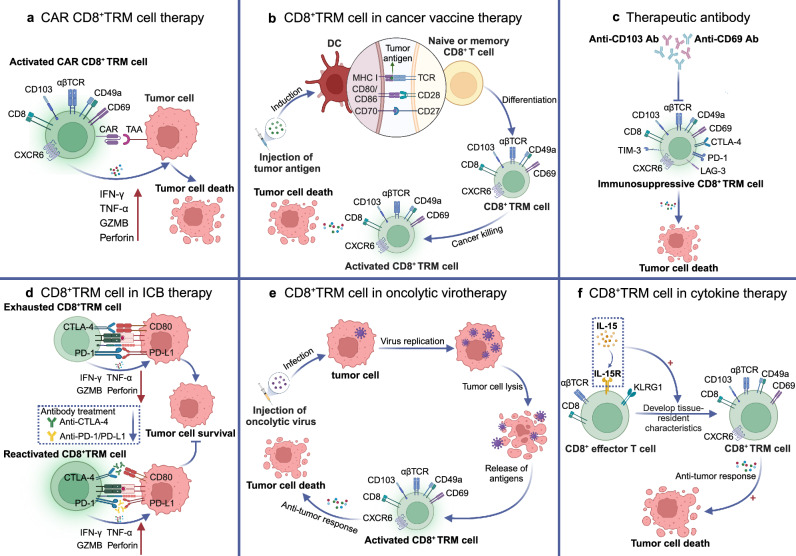
Available strategies targeting CD8^+^TRM cells in cancer immunotherapies. **a** CAR-loading renders greater cancer-killing abilities of CD8^+^TRM cells. **b** The injection of tumor antigen promotes the differentiation of memory CD8^+^T cells into TRM cells which exert cancer-killing functions. **c** The blockade of immunosuppressive CD8^+^TRM cells by applying specific antibodies can suppress their cancer-protective activities and restore anti-cancer immunity. **d** ICBs target the immune checkpoint molecules expressed on CD8^+^TRM cells to reactivate their cytotoxic effects. **e** The injection of oncolytic viruses motivates CD8^+^TRM cells to enhance specific anti-cancer immunity. **f** Cytokine therapies regulate anti-cancer-immune responses. TAA tumor-associated antigen, PD-1 programmed cell death-1, CTLA-4 cytotoxic T lymphocyte-associated antigen-4, Ab antibody, LAG-3 lymphocyte-activation gene-3, TIM-3 T cell immunoglobulin domain and mucin domain-3, ICB immune checkpoint blockade, MHC major histocompatibility complex. This figure is created with BioRender.com

Cancer immunotherapy has become one of the treatment pillars in tumors,^677^ including ICB therapies, oncolytic virus therapies, immune modulators, cancer vaccines, and adoptive transfer therapy (ACT), directly or indirectly enhancing immune-derived cancer-killing capacities.^197,678^ However, many patients do not respond well to immunotherapies, which may be ascribed to the highly heterogeneous and complex cancer-immune interactions among individuals.^679,680^ Therefore, it is important to expand our understanding of TME further and discover new targets for immunotherapies. More and more studies focus on TRICs-targeted therapy development. Here, we have summarized the feasible utilization of TRICs in cancer immunotherapies (Fig. 7).

##### Immune checkpoint blockade therapies

Immune checkpoint molecules are negative regulators of immune activation, facilitating immune homeostasis and immune escape.^681^ The roles of TRM cells in ICB immunotherapies have been investigated in melanoma, lung cancer, BC, and intrahepatic cholangiocarcinoma.^239–241^ Since many kinds of TRICs express these negative factors, they are potential biomarkers of patients’ response to ICB therapies. Especially, the number of CD8^+^TRM cells is identified as a prognostic factor for evaluating the efficacy of anti-PD-1 therapy in melanoma, TNBC, and NSCLC.^11,239,240,682^ Studies of BC explained that CD8^+^ TRM cells strengthened the local anti-tumor responses and cytotoxic capacity and restored the production of IFN-γ and TNF upon anti-CTLA-4 therapy.^241^ The interaction between IFN-γ-producing T cells and IL-12-producing DCs plays an important role in the effective response to anti-PD-1 immunotherapy,^683^ and CD49a^+^CXCR6^+^trNK cells-derived CCL5 can attract IL-12-secreted DCs and enhance intra-tumoral DC-CD8^+^T cells interaction.^14^

Apart from TRMs, other kinds of TRICs also influence the efficacy of ICB therapies. Tumor-residing MAIT cells have been found to upregulate IFN-γ production in squamous cell carcinoma following PD-1 blockade treatment, and the higher proportions of MAIT cells were positively associated with favorable responses to anti-PD-1 immunotherapy.^684,685^ PD-1^+^CD8^+^TRM cells and PD-1^+^MAIT cells interact with PD-L1^+^tissue-resident macrophages to repress immune activities,^165^ and anti-PD-1/anti-PD-L1 therapies block the interaction to reactive anti-cancer immunity.^10^ Interestingly, the combination of PD-1 blockade therapy with chemotherapy can lead to an increase of tissue-resident macrophages and TRMs infiltration in NSCLC, which provides novel biomarkers to predict immunotherapeutic efficacy.^686,687^

##### Cancer vaccine therapies

The basic principle of cancer vaccines is to improve the immune system’s ability to recognize and kill tumor cells by targeting tumor-associated antigens or tumor-specific antigens.^688^ Many studies have validated that cancer vaccine treatments can inhibit cancer progression partially by activating TRM cells. For example, intra-cervicovaginal mucosal immunization of female mouse models with human HPV vectors induces CD103^+^CD8^+^TRM cells that upregulate cytokines and perform cytotoxicity.^17,689^ And in the HPV E6/E7 oncoproteins-transformed TC-1 mouse model, the cervicovaginal vaccination with HPV E6/E7 recombinant vaccinia vaccine induces robust anti-tumor response harnessing CD103^+^CD8^+^TRM cells.^690^

Cancer vaccines also induce the generation of TRM cells in cancer.^9,691^ For example, after delivering a cancer vaccine by the intranasal mucosal route, the inducible mucosal-specific CD49a^+^CTLs suppress mucosal tumor growth.^692^ Additionally, the intranasal administration of the B subunit of Shiga toxin (STxB)-E7 vaccine elicits CD103^+^CD8^+^TRM cells in lung tissues, and these cells can strengthen the local anti-tumor response.^691^ Notably, the location of TRM cells is similarly established upon different antigen stimulations such as influenza vaccines or direct pathogen infections,^234,237^ but whether tumor antigens directly influence the development of TRM cells within TME is unclear.

Vaccine formulations, injection routes, and tissue-derived factors all influence the inducible efficacy of TRICs. Each tissue origin of cancers requires the appropriate vaccination methods to stimulate the production of TRM cells. For example, mucosal immunization is much better than intra-muscular or subcutaneous ways to elicit the formation of CD8^+^TRM cells in mucosal tumor sites.^692^ The superior effect of CD8^+^TRM cells over effector CD8^+^T cells in the same vaccination route may be due to the persistent mucosal location by which TRM cells provide rapid and long-term protection against tumors. In lung cancer, CD8^+^TRM cells induced by intranasal routes of tumor vaccines also play an indispensable and persistent role against tumor growth.^691^ Similarly, subcutaneous treatment of vaccines is effective to induce the formation of TRM cells in melanoma. In murine B16F10 melanoma models with the deletion of circulating CD8^+^T cells, intradermal vaccination motivates endogenous T cell repertoire and induces CD103^+^CD69^+^CD8^+^TRM cells that actively respond to tumor-specific (OVA) and self-antigens (GP100), and these cells also suppress tumor growth in vaccination sites and distant sites of skin, whereas the intraperitoneal route greatly reduces the anti-tumor efficacy.^693^ In the OVA-OT-1 mouse model, TILs that express VLA-1 and/or CD103 produce more IFN-γ and GZMB than integrin-negative OT-1 TILs, and the caner-repressive effects of host immunity are impaired after blocking CD103 or VLA-1.^694^ The preclinical orthotopic HCC mouse model shows that the anti-tumor effects of neoantigen vaccine are strengthened by combining with anti-PD-1 therapy, the synergy mainly mediated by CD8^+^TRM cells.^695^ Since the activation of TRM and BRM cells requires the stimulation of antigens,^56^ it is rational to prioritize the development strategies for novel cancer vaccines, with a focus on activating and inducing TRICs.

##### Adoptive cell therapies

ACT refers to transfusing specific and nonspecific immune cells with anti-tumor activity into patients’ circulation to kill tumor cells directly or motivate the anti-tumor immune responses of hosts. Many TRIC types are the candidate cell types in ACT.^12^

The technology of CAR is the most successful advance in the ACT field. General CAR-T products are made by engineering circulating T cells and play effective roles in treating hematopoietic malignancies. However, their application in solid tumors is still limited.^696^ The physical barriers and immunosuppressive cells and cytokines within TME greatly obstruct CAR-T cells from entering solid tumors and persistent functioning.^697,698^ To address this problem and improve CAR-T therapy for solid tumors, scientists fused ex vivo blood T cells with CAR engineering to generate CAR-TRM cells. Compared to CAR-T cells, the transcriptional and epigenetic profiles of CAR-TRM cells exhibit stem-like resident memory T cell characteristics. Functionally, they produce higher levels of effector cytokines and show superior resistance to exhaustion, which renders them with potent anti-tumor efficacy in both solid and hematopoietic tumor models.^699^ This innovative ACT strategy sheds light on the prospect of TRM cells in developing cancer immunotherapies.

##### Therapeutic antibodies

Targeting cancer-associated antibodies can be used to control TRIC subsistence and block their immunosuppressive effects to inhibit cancer. Here, we briefly summarize several studies that report the promising roles of therapeutic antibodies in regulating tumor immunity, including antibodies targeting receptors in tissue-resident macrophages, or basic phenotypic markers of TRICs.

In HCC, signal regulatory protein α (SIPRα) is an inhibitory receptor expressed on Kupffer cells, and its ligand CD47 is expressed on cancer cells. Inhibiting the interaction between Kupffer cells and tumor cells through blocking SIPRα-CD47 enhances phagocytosis of Kupffer cells on tumor cells.^700^

CSF plays an indispensable role in controlling the development of tissue-resident macrophages. CSF/CSF1R can trigger a cascade of intracellular tyrosine kinase activation, which promotes the survival of macrophages. Therefore, the inhibitory antibodies targeting CSF1R can alleviate the immunosuppressive effects of tissue-resident macrophages in some cancers.^701^

Many molecules participate in inducing TRM cell establishment and maintenance. For example, ICOS is a co-stimulatory receptor that plays a crucial role in inducing the formation of CD8^+^TRM cells, which will be inhibited by ICOS blockade.^702^ Moreover, the application of anti-CD45 antibodies kills tissue-resident myeloid cells in many tissues.^703^ Neutralized antibodies of CD103 and CD69 also effectively suppress the formation of immune-suppressive TRICs in some tumors.^205,627^ In short, the efficacy and kinds of therapeutic antibodies against TRICs in different diseases need further exploration.

##### Other therapies

Oncolytic viruses are genetically modified in vitro to inactivate their cytotoxicity but retain replication capacity. Targeted delivery of them to cancer cells can efficiently amplify systemic anti-tumor immunity by reversing immunosuppressive TME and triggering a cascade of proinflammatory events.^704,705^ However, the studies focusing on the application of oncolytic virotherapies functioning in a TRIC-activating/inactivating manner for cancer treatment are few. A study of pancreatic cancer showed that the administration of a variant of suratadenoturev armed with p53 (OBP-702) oncolytic virotherapy facilitated the activation and expansion of CD8^+^TRM cells and enhanced anti-tumor immunity.^706^

Some nonspecific immunotherapies also have favorable efficacy in cancer treatment such as cytokine therapy and vaccine adjuvant.^707–709^ IL-15 has been confirmed to be important for the formation of TRM cells in many tissues, and the application of IL-15 to the TME can enhance TRM cell establishment and thus promote tumor immunity.^710,711^ Adjuvants refer to substances that are usually added to vaccines to modulate immune responses, such as CpG oligodeoxynucleotide (ODN) is useful in enhancing the formation of skin-CD8^+^TRM cells and improving cancer-killing in mice B16-OVA melanoma.^712,713^ In conclusion, TRICs are promising targets for developing novel cancer immunotherapies.

### Transplantation

Organ or tissue transplantation is an important treatment strategy for some diseases. However, the allograft-induced transplant rejection greatly threatens the clinical outcomes of patients following surgery.^714^ It is required to find more ways to increase the survival of patients who have received organ transplants. TRICs persistently reside in local tissues, as well as in some transplanted solid organs.^715,716^ It is worth exploring whether those TRICs will affect organ transplant rejection.

First, TRM cells have been identified to regulate transplant rejection immunity of different organs.^715,717,718^ Donor-derived CD103^+^CD69^+^CD49a^+^TRM cells have been found to persist in lung transplants, and their numbers are positively correlated with the clinical prognosis of recipients.^719^ A study of small intestine transplantation found that CD8^+^TRM cells in transplants could reside for more than one year, and their capacity to produce cytokines and cytotoxic molecules helped defend against intestinal infections.^720^

However, TRM cells are closely related to transplant rejection. Studies of facial transplant rejection found that lymphocytes aggregated around recipients’ injured cells were donor-derived and expressed CD103 and CD69. These donor cells could attack the host skin microstructures and aggravate skin allograft rejection.^721^ A previous study indicated that the CD103^+^CD8^+^effector T cells are essential for promoting graft damage following renal transplantation.^722^ In a kidney transplant mouse model with chronic rejection features, recipient CD8^+^T cells infiltrated in the graft did not recirculate and also acquired similar phenotypic and transcriptional characteristics to TRM cells. Moreover, the depletion of graft-CD8^+^TRM cells ameliorates inflammatory damage to grafts.^723^ The unclear issue is whether the TRM cells from the recipient or the host all contribute to transplant rejection.

Except for TRM cells, tissue-resident macrophages also regulate lung transplantation.^716^ Mismatched with de novo donor-specific antibodies, donor-AMs can initiate adaptive immune responses against the transplanted lung tissues by secreting proinflammatory cytokines, resulting in obstructive airway and lung fibrosis.^724–726^

Conventional anti-rejection therapies for organ transplant patients include systemic immunosuppressants.^727^ However, the poor therapeutic effects of these drugs may be attributed to mainly targeting circulation-effector T cells, while proinflammatory TRM cells are not mentioned. Therefore, these TRM cells may serve as therapeutic targets to prolong the survival of allografts to improve clinical outcomes in the future.

## Conclusion and perspective

With the high-speed development of sequencing analysis and omics techniques, the developmental trajectories and heterogeneities of TRICs are comprehensively investigated. TRICs have been demonstrated to derive from embryonic organs or adult bone marrow, and they develop tissue-resident features at different developmental phases.^2,5,11,32,56,63–65,728^ Most TRICs can self-renew to maintain their residency in tissues, but they also depend on the supplement of blood-derived progenitors in different tissues. Tissue-resident mast cells and macrophages, trNK cells, and ILCs can derive from extramedullary hematopoiesis during adulthood, which highlights the significance of extramedullary hematopoiesis in maintaining immune cells in different tissues during adulthood, and provide us with a better understanding of the local TRIC pool composition.^68–71^ However, few studies are focusing on TRNs, and their tissue-resident mechanisms are still unclear.

Although most tissue-resident counterparts of circulating immune cells have been identified, there is no definition of whether DCs have tissue residency characteristics.^729^ Pre-DCs generate lymphoid “tissue-resident” CD8^+^DCs and CD11b^+^DCs, especially in SLOs.^730^ However, some studies indicated that DCs residing in peripheral tissues would migrate to lymphoid nodes under physiological and homeostatic conditions,^731^ so it is difficult to define the long-term “tissue residency” for DCs in different tissues.

Many factors within tissue niches participate in reshaping TRIC phenotypes and functions.^732^ Hence, the formation processes of TRICs are remarkably heterogeneous. Understanding the evolution of TRICs is important for people to develop and handle strategies of them. Interestingly, it has been found that the steady-state niches partially limit the plasticity of localized TRICs, except for endowing resident properties to preserve tissue homeostasis.^733^ Therefore, the reprogramming effects of tissue environments on TRICs are more complicated than the general imagination.

One of the major challenging tasks in TRIC studies is the identification of characteristic markers of TRICs in different tissues. Encouragingly, scRNA-seq analysis is a powerful way to find and identify the subpopulation and molecular characteristics of different TRICs. Spatial transcriptomics can help understand the compartmentalized locations of TRICs and their potential roles in controlling local immunity.

TRICs play regulator roles in different diseases. Their presence in infectious diseases is frequently correlated with favorable disease progression, suggesting their potency ability of immune defense. However, their activated immune-killing also exacerbates autoimmune diseases. Of note, the presence of most lymphoid TRICs in autoimmune diseases is induced by the previous infection. For example, the early transient viral infection results in the development of CCL5-producing TRM cells in the brain lesions of mice models with autoimmune disease. These CCL5^+^TRM cells preserve a long-term proinflammatory environment by recruiting circulating CCR5^+^autoreactive T cells and macrophages into the CNS.^734^ Lymphocytic choriomeningitis virus infection induces the formation of CNS-CD8^+^TRM cells, which can reside behind the blood-brain barrier for a long time. However, these TRM cells will attack glial cells that express the lymphocytic choriomeningitis virus glycoprotein after being infected for a while.^429^ Similarly, bacterium infection-induced TRM17 cells aggravate local renal inflammation by producing IL-17A in the experimental glomerulonephritis models.^106^ Therefore, although activated TRICs play beneficial roles in defending against infection, their long-term existence may disturb the host immune balance. This provides us with an updated understanding of TRIC functions in regulating immunity. Furthermore, monitoring the establishment and functional activities of TRICs after infections may help prevent autoimmune diseases.

Recent studies found that the infection-inducible TRICs also play a role in cancer immunity. Following influenza infection, “trained” AMs exhibit improved phagocytotic and cytotoxic functions and thus contribute to long-lasting anti-tumor immunity in a lung metastatic cancer mice model.^735^ This study provides novel insights into improving local cancer immunotherapy by trained immunity. In addition, some viral vaccines can induce TRM cells in tissues and thus enhance local anti-infection immunity.^234,581,593–596^ These vaccines may also restrain cancer development.

Many critical questions about TRICs in cancer immunity are waiting for answers: Do TRICs residing in distinct TMEs share common regulatory mechanisms of phenotypes that can be utilized for therapy development? How to activate the cancer-killing abilities of TRICs along with inactivating their immunosuppressive effects? How to evaluate the feasibility and value of TRICs as therapy and prognosis predicters? That’s not all.

Nowadays, animal models are the main methods to explore TRICs. Studies using patient samples are very few.^736^ Formulating research paradigms of TRICs based on high-throughput sequencing, flow cytometry, and tissue pathology technologies, or developing humanized mouse models may be feasible.

In conclusion, we comprehensively summarize the characteristics and functions of each TRIC type. We review their roles in physiological and pathological processes, especially their functions in regulating disease immunity. Therapies targeting TRIC regulation also show favorable outcomes in some disease treatments. Importantly, the knowledge of TRICs further expands people’s understanding of the immune system. We hope that this review will provide fundamental and comprehensive references for future research.
